# Biomass digestibility is predominantly affected by three factors of wall polymer features distinctive in wheat accessions and rice mutants

**DOI:** 10.1186/1754-6834-6-183

**Published:** 2013-12-16

**Authors:** Zhiliang Wu, Mingliang Zhang, Lingqiang Wang, Yuanyuan Tu, Jing Zhang, Guosheng Xie, Weihua Zou, Fengcheng Li, Kai Guo, Qing Li, Chunbao Gao, Liangcai Peng

**Affiliations:** 1 National Key Laboratory of Crop Genetic Improvement, Huazhong Agricultural University, Wuhan 430070, China; 2 Biomass and Bioenergy Research Centre, Huazhong Agricultural University, Wuhan 430070, China; 3 College of Plant Sciences and Technology, Huazhong Agricultural University, Wuhan 430070, China; 4 College of Life Sciences and Technology, Huazhong Agricultural University, Wuhan 430070, China; 5 College of Sciences, Huazhong Agricultural University, Wuhan 430070, China; 6 Institute of Food Crops, Hubei Academy of Agricultural Sciences, Wuhan 430064, China

**Keywords:** Cell wall, Cellulose crystallinity, Arabinose substitution degree, *p*-coumaryl alcohol proportion, Biomass digestibility, Chemical pretreatment, Wheat, Rice

## Abstract

**Background:**

Wheat and rice are important food crops with enormous biomass residues for biofuels. However, lignocellulosic recalcitrance becomes a crucial factor on biomass process. Plant cell walls greatly determine biomass recalcitrance, thus it is essential to identify their key factors on lignocellulose saccharification. Despite it has been reported about cell wall factors on biomass digestions, little is known in wheat and rice. In this study, we analyzed nine typical pairs of wheat and rice samples that exhibited distinct cell wall compositions, and identified three major factors of wall polymer features that affected biomass digestibility.

**Results:**

Based on cell wall compositions, ten wheat accessions and three rice mutants were classified into three distinct groups each with three typical pairs. In terms of group I that displayed single wall polymer alternations in wheat, we found that three wall polymer levels (cellulose, hemicelluloses and lignin) each had a negative effect on biomass digestibility at similar rates under pretreatments of NaOH and H_2_SO_4_ with three concentrations. However, analysis of six pairs of wheat and rice samples in groups II and III that each exhibited a similar cell wall composition, indicated that three wall polymer levels were not the major factors on biomass saccharification. Furthermore, in-depth detection of the wall polymer features distinctive in rice mutants, demonstrated that biomass digestibility was remarkably affected either negatively by cellulose crystallinity (CrI) of raw biomass materials, or positively by both Ara substitution degree of non-KOH-extractable hemicelluloses (reverse Xyl/Ara) and *p*-coumaryl alcohol relative proportion of KOH-extractable lignin (H/G). Correlation analysis indicated that Ara substitution degree and H/G ratio negatively affected cellulose crystallinity for high biomass enzymatic digestion. It was also suggested to determine whether Ara and H monomer have an interlinking with cellulose chains in the future.

**Conclusions:**

Using nine typical pairs of wheat and rice samples having distinct cell wall compositions and wide biomass saccharification, Ara substitution degree and monolignin H proportion have been revealed to be the dominant factors positively determining biomass digestibility upon various chemical pretreatments. The results demonstrated the potential of genetic modification of plant cell walls for high biomass saccharification in bioenergy crops.

## Background

Lignocellulosic biomass has been considered as one of the most important renewable sources for biofuels and other chemical products [1]. As the second generation of biofuels, biomass conversion into bioethanol principally involves three major steps: physical and chemical pretreatments for cell-wall disassociation, enzymatic digestion towards soluble sugar release, and yeast fermentation resulting in ethanol production [2]. However, biomass conversion is currently a costly process due to lignocellulosic recalcitrance [3,4]. Many factors such as cell wall compositions, wall polymer features, and wall network styles, determine the lignocellulosic recalcitrance [5-9]. Therefore, it becomes essential to sort out the major factors of plant cell walls that affect sugar release upon various pretreatments and sequential enzymatic hydrolysis [10].

Plant cell walls are composed primarily of cellulose, hemicelluloses, lignin and pectic polysaccharides with minor structural proteins [11]. Cellulose is one of the most abundant biopolymers in nature [12], and has a straight carbohydrate polymer chain composed of β-1, 4-glucans [13,14]. Cellulose crystallinity has been reported as a negative factor in biomass enzymatic digestibility [15-17]. Hemicelluloses are a class of heterogeneous polysaccharides, and xylans are the major components in the mature tissues of grass plants. It has been reported that hemicelluloses can negatively affect lignocellulose crystallinity for high biomass-digestibility in plants [15]. In particular, arabinose substitution degree of xylans is a positive factor in biomass enzymatic saccharification upon various chemical pretreatments in *Miscanthus*[18].

Lignin is a very stable and complex waterproofing phenolic polymer composed mainly of *p*-coumaryl alcohol (H), coniferyl alcohol (G), and sinapyl alcohol (S) [19-22]. Due to its structural diversity and heterogeneity, lignin can greatly contribute to lignocellulosic recalcitrance [23,24]. Recent reports have suggested that lignin may play dual roles in biomass enzymatic digestion, but much remains unknown in different plants [20,24,25].

Wheat and rice are the major food crops and can provide enormous biomass residues over the world [26-29]. Despite various physical, chemical, and biological pretreatments having been used for wheat and rice straw digestion [30-34], little is known about the key factors of plant cell-wall structures that greatly influence biomass enzymatic saccharification in both plants. Due to the complicated cell-wall structures and diverse biological functions, however, it remains technically difficult to find out the factors that remarkably affect biomass process [35,36]. Hence, in this study we selected the representative wheat and rice samples from the 115 wheat accessions collected in China and the 46 rice mutants generated from T-DNA insertion mutagenesis pools [14]. The selected wheat accessions and rice mutants can exhibit the characteristic cell-wall compositions that lead to sorting out three major factors of wall polymers on biomass enzymatic digestibility under various chemical pretreatments.

## Results and discussion

### Analysis of cell-wall composition in wheat and rice

Based on cell-wall composition, we selected ten wheat accessions from various genotypes and three rice mutants with different phenotypes, which were divided into three distinct groups (I, II and III) with nine pairs. In group I, three pairs (I-1, I-2, and I-3) of wheat accessions each displayed a significant difference (*P* <0.01, n = 3) of single-wall polymer (cellulose, hemicelluloses, lignin) by 30.4%, 15.1% and 27.0%, respectively (Table 1). By comparison, the other two wall polymers of each pair were only changed by less than 7% at insignificant levels (*P* >0.05, n = 3). Hence, group I can be applied to test the effect of single-wall polymer level on biomass enzymatic digestibility in wheat.

**Table 1:** Cell wall composition (% dry matter) of biomass residues in wheat samples

**Pair**	**Sample**	**Cellulose**		**Hemicelluloses**		**Lignin**	
**I**-**1**	TaLq27(H)^b^	**27.53** ± 0.98**	−**30.40**%^a^	32.63 ± 0.34	1.00%	21.76 ± 0.49*	−6.10%
	TaLq98(L)	**35.89** ± **0.70**		32.30 ± 0.11		23.08 ± 0.31	
**I**-**2**	TaLq1(H)	30.76 ± 1.35	−6.40%	**29.66** ± 0.20**	−**15.10**%	21.56 ± 0.30*	5.40%
	TaLq47(L)	32.71 ± 0.77		**34.15** ± **0.79**		20.45 ± 0.28	
**I**-**3**	TaLq107(H)	29.32 ± 0.15	−5.10%	29.68 ± 0.91	−0.80%	**19.28** ± 0.17**	−**27.00**%
	TaLq93(L)	31.59 ± 0.97		29.92 ± 0.28		**24.48** ± **0.05**	

^*^Significant difference between the two samples of each pair (*t*-test) at *P* <0.05; ^**^significant difference at *P* < 0.01 (n = 3). ^a^Percentage of the increased or decreased level between the two samples of each pair: subtraction of two samples divided by low value; ^b^samples in the pair with high (H) or low (L) biomass digestibility.

Despite group II having three typical pairs (II-1, II-2, II-3), two samples of each pair showed a very similar cell-wall composition with minor change of less than 4.1% (Table 2). Obviously, group II can be accounted for the impact of the wall polymer feature on biomass enzymatic saccharification. Furthermore, we selected three pairs of wheat and rice samples in group III (Table 3). As each pair showed a very similar wall composition between wheat and rice samples, group III can be used to perform a comparison analysis of two typical C3 grass plants in terms of their biomass digestion.

**Table 2:** **Cell wall composition** (% **dry matter**) **of biomass residues in wheat samples**

**Pair**	**Sample**	**Cellulose**		**Hemicelluloses**		**Lignin**	
**II**-**1**	TaLq1(H)^b^	30.76 ± 1.35	1.90%^a^	29.66 ± 0.20	−2.50%	21.56 ± 0.30	−4.10%
	TaLq71(L)	30.19 ± 0.38		30.40 ± 0.24		22.45 ± 0.46	
**II**-**2**	TaLq107(H)	29.32 ± 0.15	−0.20%	29.68 ± 0.91	−0.60%	19.28 ± 0.17	−2.20%
	TaLq58(L)	29.38 ± 1.15		29.85 ± 0.96		19.70 ± 1.29	
**II**-**3**	TaLq46(H)	33.76 ± 0.56	3.20%	33.70 ± 0.56	−1.30%	20.94 ± 0.33	2.40%
	TaLq47(L)	32.71 ± 0.77		34.15 ± 0.79		20.45 ± 0.28	

^a^Percentage of the increased or decreased level between the two samples of each pair: subtraction of two samples divided by low value; ^b^samples in the pair with high (H) or low (L) biomass digestibility.

**Table 3:** **Cell wall composition** (% **cell wall**) **of biomass residues in wheat and rice samples**

**Pair**	**Sample**	**Cellulose**		**Hemicelluloses**		**Lignin**	
**III**-**1**	*Osfc27*(H)^b^	35.26 ± 0.29	−3.10%^a^	38.71 ± 0.55*	5.70%	26.03 ± 0.59	−3.80%
	TaLq71(L)	36.36 ± 0.20		36.61 ± 0.52		27.03 ± 0.38	
**III**-**2**	*Osfc2*(H)	37.43 ± 0.76	0.90%	36.73 ± 0.94	−1.20%	25.84 ± 0.64	0.30%
	TaLq85(L)	37.09 ± 0.36		37.17 ± 0.51		25.75 ± 0.43	
**III**-**3**	*Osfc32*(H)	33.30 ± 0.34	−0.90%	39.27 ± 1.05	−1.40%	27.43 ± 0.75	3.20%
	TaLq27(L)	33.60 ± 0.80		39.84 ± 0.35		26.56 ± 0.79	

^*^Significant difference at pair (*t*-test) at *P* <0.05 (n = 3). ^a^Percentage of the increased or decreased level between the two samples of each pair: subtraction of two samples divided by low value; ^b^samples in the pair with high (H) or low (L) biomass digestibility.

### Determination of biomass digestibility in wheat

**Figure 1:**
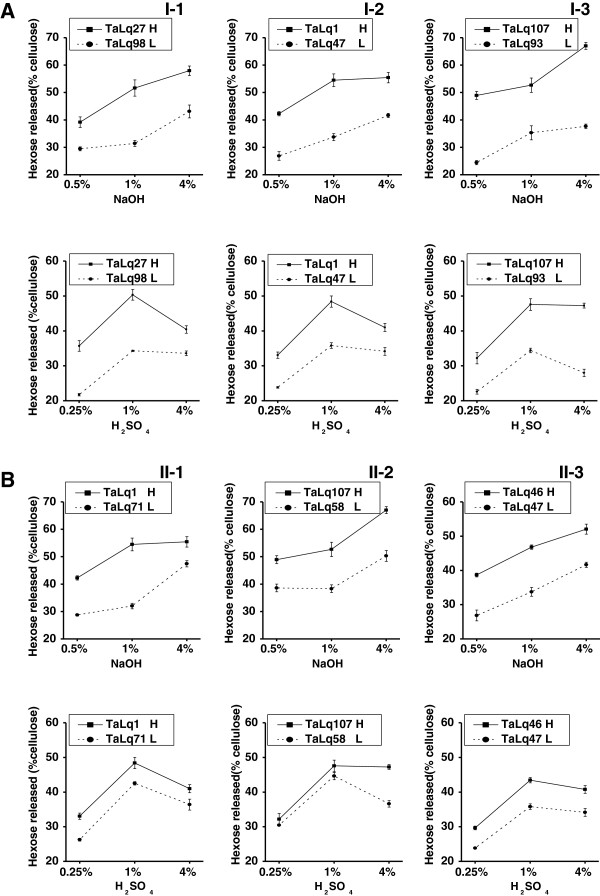
**Hexose yields (% cellulose) released from enzymatic hydrolysis after pretreatments of sodium hydroxide (NaOH) and sulfuric acid (H**_**2**_**SO**_**4**_**) at three concentrations in the representative wheat samples.** Hexose yields (% cellulose) in group I (**A**, Table 1) and group II (**B**, Table 2); the values indicate the means ± SD (n = 3); 'H' and 'L' indicate the high and low biomass digestibility.

The biomass digestibility (or saccharification) has been defined by calculating either hexose yield (% cellulose) released from hydrolysis by a crude cellulase mixture of lignocellulose after pretreatment, or total sugar (pentoses and hexoses) yield (% cell wall) from both pretreatment and enzymatic hydrolysis [15,37]. In the present work, the biomass samples were pretreated with three concentrations of sodium hydroxide (NaOH) (0.5%, 1%, and 4%) or sulfuric acid (H_2_SO_4_) (0.25%, 1%, and 4%). By comparison, all wheat samples displayed increasing hexose yields while pretreated from 0.5% to 4% NaOH, but had reducing hexose yields from 1% to 4% H_2_SO_4_ (Figure 1A, Additional file 1), which are different from the *Miscanthus* samples that remain the hexose increment with 4% H_2_SO_4_[15]. In terms of total sugar yield released, it was not much reduced from 1% and 4% H_2_SO_4_ pretreatments, but remained,rising from 0.25% to 4% H_2_SO_4_ (Additional file 2), suggesting that cellulose in wheat was partially digested by 4% H_2_SO_4_ prior to enzymatic hydrolysis. Therefore, the pretreatment of 4% H_2_SO_4_ that did not result in increased biomass digestibility (total sugar yield) in wheat, may be due to its relatively low lignin level compared with *Miscanthus*, which contained a high lignin level [15].

Due to the alternations of cell-wall composition in group I (Table 1), we found that reducing single-wall polymer levels (cellulose, hemicellulose, lignin) at three pairs (I-1, I-2, I-3) caused considerably increased biomass enzymatic digestibility by 1.2- to 2.0-fold (hexose yields) under NaOH and H_2_SO_4_ pretreatments with three concentrations (Figure 1A, Additional files 1 and 2). Notably, although the wheat sample (TaLq1) in pair I-2 showed the reduced hemicellulose level than its paired sample (Talq47) by 15.1%, the TaLq1 sample displayed a much higher biomass enzymatic saccahrification, similar to the samples in pairs I-1 and I-3 with the cellulose and lignin level changed by 30.4% and 27.0%, respectively. The results indicated that hemicelluloses, such as cellulose and lignin, may negatively affect biomass digestibility in wheat, which was similar to the findings in wood and corn [38,39], but was in contrast to *Miscanthus*[15].

To test the negative effect of three major wall polymers on biomass saccharification, we determined the wheat samples of group II, in which each pair exhibited a very similar cell-wall composition (Table 2). Like group I, the biomass enzymatic digestibility in group II was also much changed by 1.2- to 1.7-fold (hexose yields) between the two samples of each pair (II-1, II-2, II-3) after pretreatments with three concentrations of NaOH (Figure 1B, Additional files 1 and 2). By comparison, the wheat samples pretreated with H_2_SO_4_ showed relatively less change by 1.1-1.3 fold than that of NaOH. Hence, the data suggested that the cell wall composition (three major wall polymer levels) was not the major factors on biomass enzymatic digestibility in wheat, in particular, upon NaOH pretreatment.

### Comparison of biomass saccharification between wheat and rice

**Figure 2:**
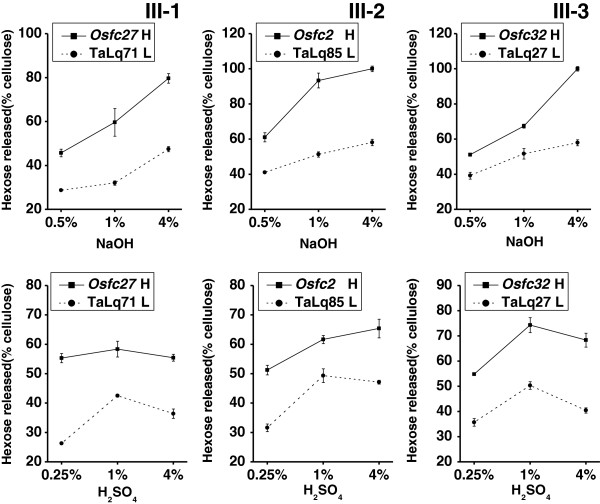
**Hexose yields (% cellulose) released from enzymatic hydrolysis after pretreatments with sodium hydroxide (NaOH) and sulfuric acid (H**_**2**_**SO**_**4**_**) at three concentrations in the representative wheat and rice samples in group III (as shown in Table**3**).** The values indicate the means ± SD (n = 3); 'H' and 'L' indicate the high and low biomass digestibility.

As the acid and alkali pretreatments could result in a different rate of biomass enzymatic digestion in group II, we further compared three pairs of wheat and rice samples (III-1, III-2, III-3) that also displayed a similar cell wall composition in group III (Table 3). Notably, all three rice mutants showed remarkably higher hexoses yields than that of their paired wheat samples by 1.2-2.1 fold after pretreatments with three concentrations of NaOH and H_2_SO_4_ (Figure 2, Additional files 1 and 2). In addition, the increased rates of hexose yields were not much different between NaOH and H_2_SO_4_ pretreatments. Therefore, it further confirmed that cell-wall composition (wall-polymer levels) was not the main factor affecting biomass enzymatic digestibility after pretreatments with both alkali and acid in wheat and rice plants. In other words, the data hinted that cell-wall structures (wall-polymer features) should mainly affect biomass saccharification upon chemical pretreatments.

Furthermore, the three rice mutants exhibited much higher hexose yields or total sugar yields than that of all ten wheat samples under NaOH and H_2_SO_4_ pretreatments (Additional files 1 and 2). For instance, pretreated with 1% NaOH, three rice mutants (*Osfc27*, *Osfc2* and *Osfc32*) respectively displayed hexose yields (% cellulose) of 59.6%, 93.4%, and 67.4% or total sugar yields (% cell wall) of 72.3%, 76.9%, and 74.2%, whereas ten wheat samples had hexose yields ranging from 31.4% to 54.5% or total sugar yields ranging from 37.8% to 50.8%. Similarly, the *Osfc32* mutant pretreated with 1% H_2_SO_4_ displayed the highest hexose and total sugar yields at 74.3% and 86.4%, but Talq27 and Talq107 accessions had the highest hexose or total sugar yields at 50.3% or 55.2%, respectively.

### Observation of biomass residue surface

**Figure 3:**
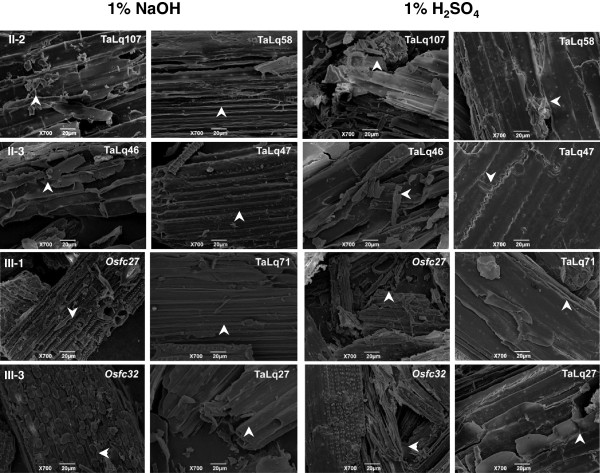
**Scanning electron microscope images of biomass residues obtained from pretreatment with 1% sodium hydroxide (NaOH) or 1% sulfuric acid (H**_**2**_**SO**_**4**_**), and sequential enzymatic hydrolysis.** Wheat and rice samples (TaLq107, TaLq46, *Osfc27*, and *Osfc32*) with a relatively higher biomass digestibility showing a relatively coarse surface indicated by the arrow, and wheat sample (TaLq58, TaLq47, TaLq71, TaLq27) displaying a flat face.

Scanning electron microscopy was applied for observation of biomass residue surface after 1% NaOH and 1% H_2_SO_4_ pretreatments and sequential enzymatic hydrolysis of four representative pairs (II-2, II-3, III-1, III-3) of wheat and rice samples (Figure 3). The samples (Talq107, Talq46, *Osfc27*, and *Osfc32*) with high biomass-digestibility displayed a coarse biomass residue surface, whereas their paired samples (Talq58, Talq47, Talq71, and Talq27) exhibited a relatively smooth face, similar to observations in *Miscanthus*[15,18]. In particular, two rice mutants showed much rougher surfaces than that of all six wheat samples, consistent with their quite different biomass digestion rates (Additional files 1 and 2). Hence, the rough face of biomass residue should be due to a relatively effective enzymatic hydrolysis after chemical pretreatment. In addition, as the four pairs each had a similar cell-wall composition, it confirmed that the biomass residue surface may mainly be affected by wall polymer features.

### Effects of wall-polymer features on biomass enzymatic digestion

**Figure 4:**
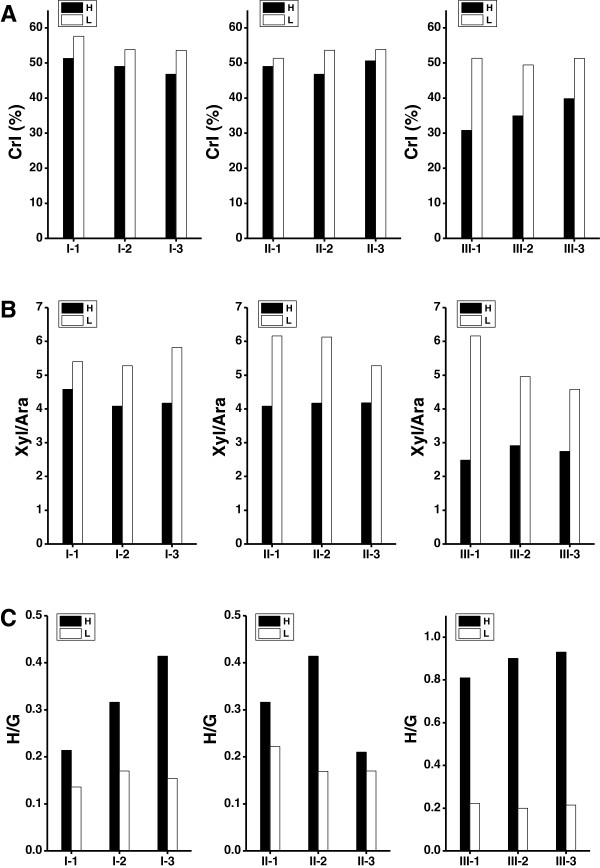
**Comparison of three wall-polymer features (crystalline index (CrI), xylose/arabinose (Xyl/Ara), *p*-coumaryl alcohol/coniferyl alcohol (H/G)) between two samples of each pair in wheat accessions and rice mutants. (A)** CrI of raw material, **(B)** Xyl/Ara of non-potassium hydroxide (KOH)-extractable hemicelluloses, **(C)** H/G of KOH-extractable lignin. 'H' and 'L' indicate the high and low biomass digestibility.

To confirm the effects of cell-wall structures on biomass enzymatic digestion in wheat and rice, we analyzed three major wall-polymer features including cellulose crystalline index (CrI) (Additional file 3), monosaccharide composition of hemicelluloses (Additional file 4), and monomer constitution of lignin (Additional file 5). Among the nine pairs in three groups, the biomass samples having relatively high biomass digestibility showed much lower cellulose CrI values than that of their paired samples with low biomass saccharification (Figure 4A), indicating that the cellulose CrI was the negative factor on biomass digestibility in wheat and rice. Notably, three rice mutants displayed extremely lower CrI values at 30.8%, 35.0% and 39.9% than that of all ten wheat accessions ranging from 46.8% to 57.6% (Additional file 3). These results were consistent with their quite different biomass enzymatic digestibility.

With regard to monosaccharide composition of hemicelluloses, we found that arabinose (Ara) and xylose (Xyl) covered more than 95% of total monosaccharides (Additional file 4), indicating that xylans are the major hemicelluloses in both wheat and rice plants. As the Xyl/Ara ratio has been applied as a negative indicator for the Ara substitution degree of xylans in *Miscanthus*[17], we calculated the Xyl/Ara values of both potassium hydroxide (KOH)-extractable and non-KOH-extractable hemicelluloses. In general, the non-KOH-extractable hemicelluloses contained the largest difference in Xyl/Ara values between two samples of each pair (ranging from −18.0% to −148.1%), compared with the KOH-extractable hemicelluloses (−1.6% to −75.9%) and total hemicelluloses (−1.2% to −79%) (Figure 4B, Additional file 4). Like cellulose CrI, the biomass samples with high biomass digestibility had much lower Xyl/Ara values than that of their paired samples in the non-KOH-extractable hemicelluloses, in particular in three pairs of group III. Hence, the data indicated that Ara substitution degree of the non-KOH-extractable xylans positively affected biomass enzymatic digestibility in wheat and rice, similar to the role of Xyl/Ara in *Miscanthus*[17].

In terms of three monolignin (H, G, S) constitution, we calculated the ratios (H/G, H/S, S/G) in the KOH-extractable and non-KOH-extractable lignins (Additional file 5). By comparison, H/G values of the KOH-extractable lignin were much more alternated between two samples of each pair than any other monolignin ratios (Figure 4C, Additional file 5). Unlike cellulose CrI or hemicellulose Xyl/Ara, the biomass samples with high biomass-digestibility displayed extremely higher H/G values than that of their paired samples in the KOH-extractable lignin, in particular in three pairs of group III. Thus, this is the first time report of H/G (or the H proportion) as a positive factor in biomass enzymatic saccharification in wheat and rice. On the other hand, although S/G has been reported as a negative factor in *Miscanthus* and other plants [15,40-42], the nine pairs of biomass samples exhibited an inconsistent and small alternation of S/G, suggesting that the S/G was not the major factor in wheat and rice.

In summary, three pairs of wheat and rice samples in group III exhibited much more alternations of CrI, Xyl/Ara and H/G values than that of the other six pairs of wheat samples in groups I and II (Figure 4), consistent with the remarkably high biomass-digestibility in rice mutants.

### Correlation among wall-polymer features and biomass digestibility

**Figure 5:**
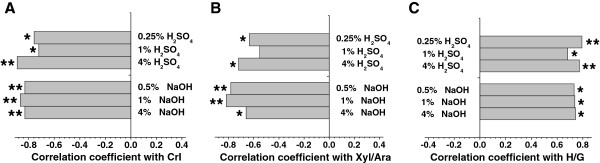
**Correlation analysis between three wall-polymer features (crystalline index (CrI), xylose/arabinose (Xyl/Ara) and *p*-coumaryl alcohol/coniferyl alcohol (H/G)) and hexose yield (% cellulose) released from enzymatic hydrolysis after sodium hydroxide (NaOH) and sulfuric acid (H**_**2**_**SO**_**4**_**) pretreatments in wheat (n = 10). (A)** CrI of raw material, **(B)** Xyl/Ara of non-potassium hydroxide (KOH)-extractable hemicelluloses, **(C)** H/G of KOH-extractable lignin. ^*^P < 0.05 for correlation coefficient values; ^**^P <0.01for correlation coefficient values.

As aforementioned, biomass enzymatic digestibility was largely affected by three major factors including cellulose CrI of raw biomass material, Xyl/Ara of non-KOH-extractable hemicelluloses and H/G of KOH-extractable lignin. To confirm the findings, we performed an in-depth correlative analysis between three wall-polymer features and hexose yields released from enzymatic hydrolysis after pretreatments with three concentrations of NaOH and H_2_SO_4_ in the wheat and rice samples examined (Figure 5). Significantly, both cellulose CrI and hemicellulosic Xyl/Ara were negatively correlated with the hexose yields from various pretreatments, at *P* <0.05 or 0.01 (n = 10), except 1% H_2_SO_4_ for Xyl/Ara (Figure 5A and 5B). By contrast, the lignin H/G showed a significant positive correlation with hexose yields from various pretreatments (*P* <0.05 or 0.01, n = 10) (Figure 5C). The insignificant correlation from 1% H_2_SO_4_ pretreatment may be due to the limited sample numbers of wheat examined in this study. The results reconfirmed that these three wall polymers significantly affected biomass digestibility under various chemical pretreatments.

**Figure 6:**
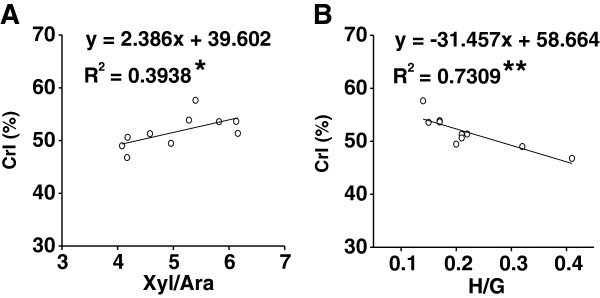
**Correlation analysis of crystalline index (CrI), xylose/arabinose (Xyl/Ara) and *p*-coumaryl alcohol/coniferyl alcohol (H**/**G) in wheat (n = 10). (A)** Xyl/Ara of non-potassium hydroxide (KOH)-extractable hemicelluloses, **(B)** H/G of KOH-extractable lignin. *P* < 0.05 for correlation coefficient values; ^**^P < 0.01 for correlation coefficient values.

To find out the associations among three major wall-polymer features, we also calculated their correlation coefficients in this work (Figure 6). As a result, the cellulose CrI showed a significantly positive correlation with the Xyl/Ara of non-KOH-extractable hemicelluloses at *P* <0.05 (Figure 6A). It suggests that the branched Ara of xylans may be interlinked with β-1, 4-glucan chains by hydrogen bonds that can reduce cellulose crystallinity in wheat and rice, as in *Miscanthus* as discussed [18]. Notably, there was even much higher negative correlation between cellulose CrI and H/G of KOH-extractable lignin with an *R*^2^ value of 0.73 (*P* <0.01) (Figure 6B). Although it has been interpreted that lignin has an indirect impact on cellulose crystallinity by its interaction with hemicellulose in *Miscanthus*[15], our data suggested that H monomer may interlink with β-1, 4-glucan chains rather than hemicellulose, which leads to the reduced cellulose crystallinity in wheat and rice. However, the Ara and H-monomer interlinking with cellulose chains has yet to be tested in the future.

### Potential modification of plant cell walls for high biomass digestibility

**Figure 7:**
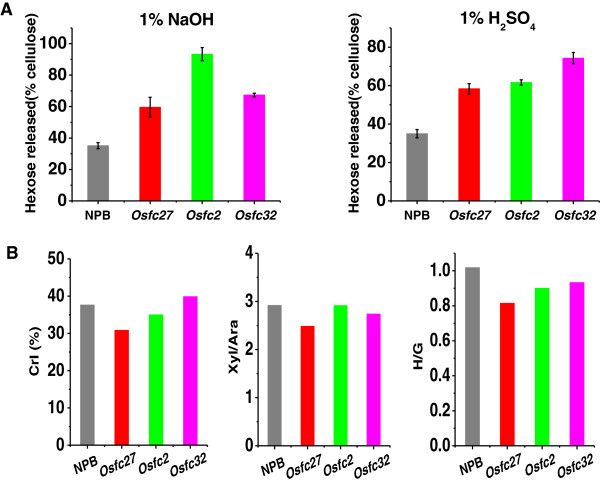
**Comparison of hexose yields and cell-wall features between wild-type, nippobare (NPB) and three mutants of rice. (A)** Hexose yield (% cellulose) released from enzymatic hydrolysis after 1% sodium hydroxide (NaOH) or 1% sulfuric acid (H_2_SO_4_) pretreatment, **(B)** three wall polymer features (crystalline index (CrI) of raw material, xylose/arabinose (Xyl/Ara) of non-potassium hydroxide (KOH)-extractable hemicelluloses, *p*-coumaryl alcohol/coniferyl alcohol (H/G) of KOH-extractable lignin).

As wheat and rice are the most important food resources with a high amount of biomass residues, they are currently considered as potential bioenergy crops. However, plant biomass constitutes numerous different cell types with diverse wall components, and thus, identifying desirable cell walls for high biomass digestibility is quite difficult [35,36]. Specifically, due to the complicated structures and diverse functions of plant cell-walls [36], determining the effects of the three major wall polymers (cellulose, hemicellulose, and lignin) on biomass digestion is not simple. In this study, analysis of nine typical pairs of wheat and rice samples with distinct cell-wall composition/structure and wide biomass saccharification rates, has resulted in identification of three major wall-polymer features (CrI, Xyl/Ara, H/G), other than wall-polymer levels, that predominately affect biomass digestibility. Notably, to our knowledge, this is the first report of the positive effect of H/G on biomass digestion, and of three major wall-polymer features in wheat and rice. Although cellulose CrI is a negative factor, both Ara substitution degree (reverse Xyl/Ara) and H/G can positively affect biomass digestibility by reducing cellulose crystallinity in wheat and rice. Thus, this study has principally indicated the potential approaches of cell-wall modifications for high biomass-digestibility by increasing either non-KOH-extractable Ara substitution degree or KOH-extractable H-monomer proportion, or both factors, in wheat and rice crops. There are several advantages in support of the cell-wall modifications in this study: (1) non-KOH-extractable xylans cover less than 16% of total hemicelluloses in rice (Additional file 6), indicating a minor modification of hemicellulose; (2) KOH-extractable H monomer contains about 13% of total three monolignins in wheat (Additional file 7); (3) despite the three rice mutants all having much higher biomass digestibility than the wild-type rice, nipponbare (NPB) (Figure 7A), they showed distinct changes in the three major wall-polymer features (Figure 7B). Compared with NPB, the *Osfc27* mutant displayed reduced values of CrI, Xyl/Ara and H/G, whereas *Osfc2* and *Osfc32* respectively showed reductions of only two features (CrI and H/G; Xyl/Ara and H/G), suggesting multiple alternations of three major wall-polymer features; (4) three mutants had a normal growth with grain yields and biomass products similar to the wild type (data not shown), indicating the possibility of minor modification of plant cell-walls in bioenergy crops; (5) KOH extraction of biomass residues was performed under mild conditions (4 M KOH with sodium borohydride for 1 h at 25°C) and the non-KOH-extractable residues should thus relatively remain a native structural state, suggesting that Ara substitution degree of non-KOH-extractable xylans is a native (true) factor for genetic modification of plant cell walls. Genetic modification of plant cell-walls is considered as a promising solution to lignocellulosic recalcitrance for high biofuel productions [4]. Hence, this study provides new insights into genetic engineering of the major cell-wall polymer features in energy crop breeding.

## Conclusions

The three main wall-polymer features, including cellulose CrI, non-KOH-extractable Xyl/Ara of hemicellulose, and KOH-extractable H/G of lignin, rather than cell-wall composition (wall-polymer levels), have been revealed as predominant factors in biomass enzymatic digestibility upon various chemical pretreatments. It has been indicated that either Ara substitution degree or H monolignin proportion negatively affects CrI for high biomass-saccharification. The results also suggest the potential of cell-wall modifications for biofuel production in wheat, rice and other bioenergy crops.

## Methods

### Plant samples

The 115 wheat accessions were provided by Hubei Agricultural Science Academy in Hubei Province, China. They represent a diversity of winter wheat germplasm adapted to growth in Yangzi River regions in central China. All the wheat samples were collected from Hubei experimental fields in the growing season of 2010. A total of 46 homozygous rice mutants with fragile or high culm phenotypes were originally selected from T-DNA mutant pools as described by Xie *et al*. 2013 [14], and their mature culms tissues were collected from the Wuhan experimental fields in 2009 and 2010. The collected samples were dried at 50°C after inactivation at 105°C for 20 minutes. The dried tissues were ground into powder through a 40-mesh screen and stored in a dry container until use.

### Plant cell-wall fractionation

The plant cell-wall fractionation method was used to extract cellulose and hemicelluloses, as described by Peng *et al*., 2000 [43], and Xu *et al*., 2012 [15] with minor modification. The soluble sugar, lipids, starch and pectin of the samples were successively removed by potassium phosphate buffer (pH 7.0), chloroform-methanol (1:1, v/v), dimethylsulphoxide (DMSO)-water (9:1, v/v) and 0.5% (w/v) ammonium oxalate. The remaining pellet was extracted with 4 M KOH with 1.0 mg/mL sodium borohydride for 1 h at 25°C, and the combined supernatant with two parallels; one parallel was neutralized, dialyzed and lyophilized as KOH-extractable hemicelluloses monosaccharides, and one parallel was collected for determination of free pentoses as the KOH-extractable hemicelluloses. For the remaining two parallel non-KOH-extractable residues, one parallel was sequentially extracted with trifluoroacetic acid (TFA) for monosaccharides, and one parallel was further extracted with H_2_SO_4_ (67%, v/v) for 1 h at 25°C, and the supernatants were collected for determination of free hexoses and pentoses as total cellulose and non-KOH-extractable hemicelluloses. All experiments were carried out in biological triplicate.

### Colorimetric assay of hexoses and pentoses

The UV–VIS spectrometer (V-1100D, Shanghai MAPADA Instruments Co., Ltd. Shanghai, China) was used for the absorbance reading. Hexoses were detected using the anthrone/H_2_SO_4_ method [44], and pentoses were tested using the orcinol/HCl method [45]. Anthrone was purchased from Sigma-Aldrich Co. LLC., and ferric chloride and orcinol were obtained from Sinopharm Chemical Reagent Co., Ltd. The standard curves for hexoses and pentoses were drawn using d-glucose and d-xylose as standards (purchased from Sinopharm Chemical Reagent Co., Ltd.) respectively. The total sugar yield from pretreatment and enzymatic hydrolysis was subject to the sum total of hexoses and pentoses. High pentose levels can affect the absorbance reading at 620 nm for hexose content using the anthrone/H_2_SO_4_ method, so deduction from the pentose reading at 660 nm was carried out for final hexose calculation. A series of xylose concentrations were analyzed for plotting the standard curve referred to for the deduction, which was verified by gas chromatography–mass spectrometry (GC-MS) analysis. All experiments were carried out in biological triplicate.

### Hemicellulose monosaccharide determination by GC-MS

Determination of hemicellulose monosaccharides was described by Li *et al*., 2013 [18]. The combined supernatants from 4 M KOH fraction were dialyzed for 36 h after neutralization with acetic acid, and the sample from the dialyzed KOH-extractable supernatant or the non-KOH-extractable residue was hydrolyzed by 2 M TFA for free monosaccharide release in a sealed tube at 121°C in an autoclave for 1 h. *Myo*-inositol (200 μg) was added as the internal standard for GC-MS (SHIMADZU GCMS-QP2010 Plus).

GC-MS was performed using the following: analytical conditions: Restek Rxi-5 ms, 30 m × 0.25 mm ID × 0.25 um df column; carrier gas: helium; injection method: split; injection port: 250°C; interface: 250°C; injection volume: 1.0 μL; temperature program: from 155°C (held for 23 minutes) to 200°C (held for 5 minutes) at 3.8°C/minute, then from 200°C to 300°C (held for 2 minutes) at 20°C/minute; ion source temperature: 200°C; ACQ mode: SIM. The mass spectrometer was operated in the EI mode with ionization energy of 70 ev. Mass spectra were acquired with full scans based on the temperature program from 50 to 500 m/z in 0.45 s. Calibration curves of all analytes routinely yielded correlation coefficients of 0.999 or higher.

### Total lignin assay

Total lignin content was determined by the two-step acid hydrolysis method according to Laboratory Analytical Procedure of the National Renewable Energy Laboratory. The lignin includes acid-insoluble and -soluble lignin. The acid-insoluble lignin was calculated gravimetrically after correction for ash, and the acid-soluble lignin was measured by UV spectroscopy.

For acid-insoluble lignin determination, a 0.5-g sample was recorded as W1. The sample was extracted with benzene-ethanol (2:1, v/v) in a Soxhlet for 4 h, and then air-dried in a hood overnight. The sample was hydrolyzed with 10 mL 72% H_2_SO_4_ (v/v) in a shaker at 30°C for 1.5 h. After hydrolysis, the acid was diluted to a concentration of 2.88%, and then placed in the autoclave for 1 h at 121°C (15 psi). The autoclaved hydrolysis solution was vacuum-filtered through the previously weighed filtering crucible. The filtrate was captured in a filtering flask for acid-soluble lignin. The lignin was washed free of acid with hot distilled water and the crucible and acid-insoluble residue was dried in an oven at 80°C until constant weight was achieved. Then, the samples were removed from the oven and cooled in a dry container. The weight of the crucible and dry residue was recorded to the nearest 0.1 mg (W2). Finally the dried residue was ashed in the muffle furnace at 200°C for 30 minutes and at 575°C for 4 h. The crucibles and ash were weighed to the nearest 0.1 mg and we recorded the weight (W3). The acid-insoluble lignin (AIL) of the original sample was calculated as follows:

$$\mathrm{AIL}\;\left(\%\right)=\left(W2–W3\right)\times 100/W1\%.$$

Each sample was tested in biological triplicate. For the acid-soluble lignin determination, the acid-soluble lignin was solubilized during the hydrolysis process, and was measured by UV spectroscopy. The hydrolysis liquor obtained previously was transferred into a 250-mL volumetric flask and brought up to 250 mL with 2.88% sulfuric acid. The absorbance of the sample was read at 205 nm using UV–vis spectroscopy (Beckman Coulter Inc., Du800), and 2.88% sulfuric acid was used as blank. The method of calculation for the amount of acid-soluble lignin was as follows:

$$\mathrm{ASL}\;\left(\%\right)=\left(A\times D\times V/1000\times K\times W1\right)\times 100\;\%$$

Where A is the absorption value, D is the dilution ratio of the sample, and K (the absorptivity constant) = 110 L/g/cm.

$$\mathrm{Total}\;\mathrm{lignin}\;\left(\%\right)=\mathrm{ASL}\%+\mathrm{AIL}\%.$$

All experiments were carried out in triplicate.

### Lignin monomer detection by high performance liquid chromatography (HPLC)

Lignin monomer determination was as described by Xu *et al*., 2012 [15]. The standard chemicals, *p*-Hydroxybenzaldehyde (H), vanillin (G) and syringaldehyde (S) were purchased from Sinopharm Chemical Reagent Co., Ltd. The sample was extracted with benzene-ethanol (2:1, v/v) in a Soxhlet for 4 h, and the remaining pellet was collected as cell-wall residue (CWR). The procedure for nitrobenzene oxidation of lignin was conducted as follows: 0.05 g CWR was added with 5 mL 2 M NaOH and 0.5 mL nitrobenzene, and a stir bar was put into a 25-mL Teflon gasket in a stainless steel bomb. The bomb was sealed tightly and heated at 170°C (oil bath) for 3.5 h and stirred at 20 rpm. Then, the bomb was cooled with cold water. The chromatographic internal standard (ethyl vanillin) was added to the oxidation mixture. This alkaline oxidation mixture was washed three times with 30 mL CH_2_C1_2_/ethyl acetate mixture (1:1, v/v) to remove nitrobenzene and its reduction by-products. The alkaline solution was acidified to pH 3.0 to 4.0 with 6 M HCl, and then extracted with CH_2_CI_2_/ethyl acetate (3 × 30 mL) to obtain the lignin oxidation products, which were in the organic phase. The organic extracts were evaporated to dryness under reduced pressure at 40°C. The oxidation products were dissolved in 10 mL chromatographic pure methanol.

For HPLC analysis the solution was filtered with a membrane filter (0.22 μm). Then, 20 μL solution was injected into the HPLC (Waters 1525 HPLC) column Kromat Universil C18 (4.6 mm × 250 mm, 5 μm) operating at 28°C with CH_3_OH:H_2_O:HAc (25:74:1, v/v/v) carrier liquid (flow rate: 1.1 mL/minute). Calibration curves of all analytes routinely yielded correlation coefficients 0.999 or higher, and the detection of the compounds was carried out with a UV-detector at 280 nm.

### Detection of cellulose crystallinity

The X-ray diffraction method was described by Zhang *et al*., 2013 [17] for detection of cellulose CrI using the Rigaku-D/MAX instrument (Uitima III, Japan). The well-mixed powders of biomass samples were laid on the glass sample-holder (35 × 50 × 5 mm) and were analyzed under plateau conditions. Ni-filtered Cu Kα radiation (λ = 0.154056 nm) was generated at a voltage of 40 kV and a current of 18 mA, and scanned at a speed of 0.0197°/s from 10° to 45°. The CrI was estimated using the intensity of the 200 peak (I_200_, θ = 22.5°) and the intensity at the minimum between the 200 and 110 peaks (I_am_, θ = 18.5°) as follow:

$$\mathrm{CrI}=100\times \left(I_{200}–I_{\mathrm{am}}\right)/I_{200}.$$

I_200_ represents both crystalline and amorphous materials while Iam represents amorphous material. The standard error of the CrI method was detected at ± 0.05 to approximately 0.15 using five representative samples in triplicate.

### Scanning electron microscopy (SEM) observations

The well-mixed biomass powder samples were pretreated with 1% NaOH or 1% H_2_SO_4_, and hydrolyzed with the mixed cellulases. The remaining residues were washed with distilled water until the pH was 7.0. The surface morphology of the sample was sputter-coated with gold and observed by SEM (SEM JSM-6390/LV, Hitachi, Tokyo, Japan) as described by Xu *et al*., 2012 [15]. Each sample was observed 5 to 10 times and the representative image was used in this study.

### Biomass pretreatment

Chemical pretreatments were performed as previously described by Huang *et al*., 2012 [37] with minor modification. For H_2_SO_4_ pretreatment, the well-mixed powder of the biomass sample (0.3 g) was added with 6 mL H_2_SO_4_ at three concentrations (0.25%, 1%, 4%, v/v). The tube was sealed and heated at 121°C for 20 minutes in an autoclave (15 psi) after mixing well. Then, the tube was shaken at 150 rpm for 2 h at 50°C, and centrifuged at 3,000 *g* for 5 minutes. The pellet was washed three times with 10 mL distilled water, and stored at −20°C for enzymatic hydrolysis. All supernatants were collected for determination of total sugars (pentoses and hexoses) released from acid pretreatment, and samples with 6 mL distilled water were shaken for 2 h at 50°C as the control. All samples were carried out in biological triplicate.

For NaOH pretreatment, the well-mixed powder of the biomass sample (0.3 g) was added with 6 mL NaOH at three concentrations (0.5%, 1%, 4%, w/v). The tube was shaken at 150 rpm for 2 h at 50°C, and centrifuged at 3,000 *g* for 5 minutes. The pellet was washed three times with 10 mL distilled water, and stored at −20°C for enzymatic hydrolysis. All supernatants were collected for determination of total sugars released from alkali pretreatment, and samples with 6 mL distilled water were shaken for 2 h at 50°C as the control. All samples were carried out in biological triplicate.

### Enzymatic hydrolysis

The remaining residues from various pretreatments were washed twice with 10 mL distilled water, and once with 10 mL of mixed-cellulase reaction buffer (0.2 M acetic acid-sodium acetate, pH 4.8). The washed residues were added with 6 mL (1.6 g/L) of mixed cellulases containing β-glucanase (≥2.98 × 10^4^ U), cellulase (≥298 U) and xylanase (≥4.8 × 10^4^ U) from Imperial Jade Bio-technology Co., Ltd) at 0.16% (w/w) concentration for H_2_SO_4_- and NaOH- pretreated samples. During the enzymatic hydrolysis, the samples were shaken at 150 rpm at 50°C for 48 h. After centrifugation at 3,000 *g* for 10 minutes, the supernatants were collected to determine the amounts of pentose and hexose released from enzymatic hydrolysis. The samples with 6 mL of reaction buffer were shaken for 48 h at 50°C as the control. All samples were carried out in biological triplicate.

### Statistical calculation of correlation coefficients

Correlation coefficients were generated by performing Spearman rank correlation analysis for all pairs of measured traits across the whole population. This analysis used average values calculated from all original determinations for a given traits pair.

## Abbreviations

CrI: crystalline index; Ara: arabinose; Xyl: xylose; Rha: rhamnose; Fuc: fucose; Man: mannose; Glu: glucose; Gla: galactose; H: *p*-coumaryl alcohol; H2SO4: sulfuric acid; G: coniferyl alcohol; KOH: potassium hydroxide; S: sinapyl alcohol; AIL: acid-insoluble lignin; ASL: acid-soluble lignin; NaOH: sodium hydroxide; NPB: nipponbare; CWR: cell wall residue; DMSO: dimethylsulphoxide; TFA: trifluoroacetic acid; GC-MS: gas chromatography-mass spectrometer; HPLC: high performance liquid chromatography; SEM: scanning electron microscopy.

## Competing interests

The authors declare that they have no competing interests.

## Authors’ contributions

ZW and MZ completed the major experiments. YT, JZ and QL participated in chemical analysis. GX, WZ, FL and KG selected and characterized the rice mutants. CG completed wheat sample collection. LW designed the wheat experiments and analyzed rice mutants. LP designed the project, supervised the experiments, interpreted the data and finalized the manuscript. All authors read and approved the final manuscript.

## Supplementary Material

Additional file 1: Table S1 Hexose yield (% cellulose) released from enzymatic hydrolysis after pretreatment. Exhibited are comparisons of biomass enzymatic digestibility (hexose yield) after sodium hydroxide (NaOH) and sulfuric acid (H_2_SO_4_) pretreatments with three concentrations, among a total of nine pairs of wheat and rice samples. Click here for file

Additional file 2: Table S2 Total sugar yield (% cell wall) released from both enzymatic hydrolysis and pretreatment. Exhibited are comparisons of biomass enzymatic digestibility (total hexose and pentose yield) upon sodium hydroxide (NaOH) and sulfuric acid (H_2_SO_4_) pretreatments with three concentrations, among a total of nine pairs of wheat and rice samples. Click here for file

Additional file 3: Table S3 The crystalline index (CrI) of raw materials in wheat and rice samples. Displayed are comparisons of CrI values among a total of nine pairs of wheat and rice samples. Click here for file

Additional file 4: Table S4 Monosaccharide composition of hemicelluloses. Displayed are comparisons of monosaccharide compositions in the potassium hydroxide (KOH)-extractable and non-KOH-extractable hemicelluloses among a total of nine pairs of wheat and rice samples. Click here for file

Additional file 5: Table S5 Ratios of three monolignins. Displayed are comparisons of three monomer ratios in the potassium hydroxide (KOH)-extractable and non-KOH-extractable lignin among a total of nine pairs of wheat and rice samples. Click here for file

Additional file 6: Table S6 Variation of two types of hemicellulose (μmol/g dry matter). Exhibited are proportions between the potassium hydroxide (KOH)-extractable and non-KOH-extractable hemicelluloses in the representative wheat (n = 10) and rice (n = 3) samples. Click here for file

Additional file 7: Table S7 Variation of two types of lignin (μmol/g dry matter). Exhibited proportions between the potassium hydroxide (KOH)-extractable and non-KOH-extractable lignin in the representative wheat (n = 10) and rice (n = 3) samples. Click here for file
