# Supplementary material for: Biomass digestibility is predominantly affected by three factors of wall polymer features distinctive in wheat accessions and rice mutants
Source: Biotechnol Biofuels. 2013 Dec 16;6:183. doi: 10.1186/1754-6834-6-183 (PMC3878626; doi:10.1186/1754-6834-6-183)
Supplement: Additional file 1: Table S1 — Hexose yield (% cellulose) released from enzymatic hydrolysis after pretreatment. Exhibited are comparisons of biomass enzymatic digestibility (hexose yield) after sodium hydroxide (NaOH) and sulfuric acid (H2SO4) pretreatments with three concentrations, among a total of nine pairs of wheat and rice samples. [file 1754-6834-6-183-S1.pptx]

## Slide 1
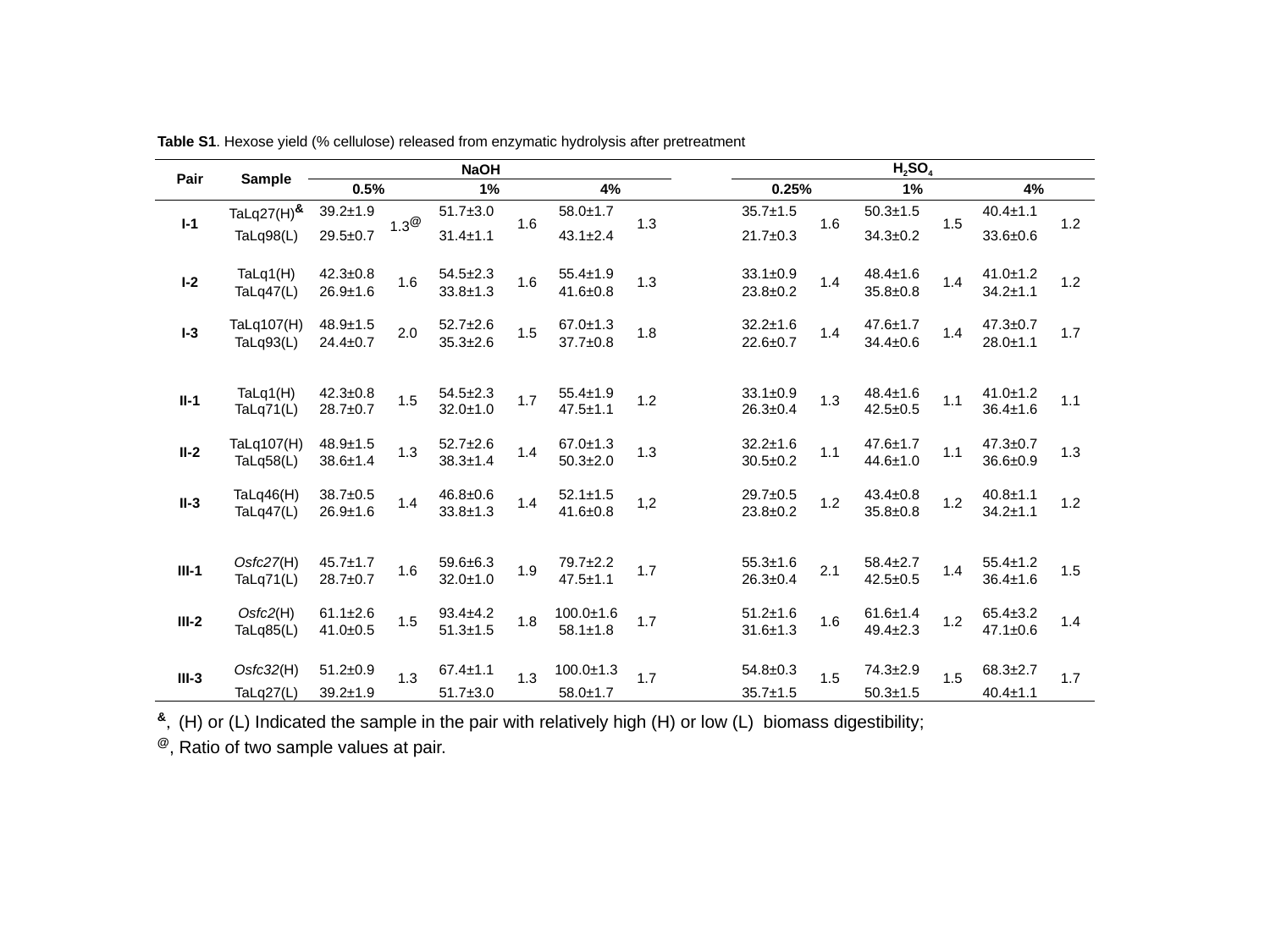

# Table S1. Hexose yield (% cellulose) released from enzymatic hydrolysis after pretreatment
| Pair | Sample | NaOH | | | | | | | H2SO4 | | | | | |
| --- | --- | --- | --- | --- | --- | --- | --- | --- | --- | --- | --- | --- | --- | --- |
| | | 0.5% | | 1% | | 4% | | | 0.25% | | 1% | | 4% | |
| I-1 | TaLq27(H)& | 39.2±1.9 | 1.3@ | 51.7±3.0 | 1.6 | 58.0±1.7 | 1.3 | | 35.7±1.5 | 1.6 | 50.3±1.5 | 1.5 | 40.4±1.1 | 1.2 |
| | TaLq98(L) | 29.5±0.7 | | 31.4±1.1 | | 43.1±2.4 | | | 21.7±0.3 | | 34.3±0.2 | | 33.6±0.6 | |
| | | | | | | | | | | | | | | |
| I-2 | TaLq1(H) | 42.3±0.8 | 1.6 | 54.5±2.3 | 1.6 | 55.4±1.9 | 1.3 | | 33.1±0.9 | 1.4 | 48.4±1.6 | 1.4 | 41.0±1.2 | 1.2 |
| | TaLq47(L) | 26.9±1.6 | | 33.8±1.3 | | 41.6±0.8 | | | 23.8±0.2 | | 35.8±0.8 | | 34.2±1.1 | |
| | | | | | | | | | | | | | | |
| I-3 | TaLq107(H) | 48.9±1.5 | 2.0 | 52.7±2.6 | 1.5 | 67.0±1.3 | 1.8 | | 32.2±1.6 | 1.4 | 47.6±1.7 | 1.4 | 47.3±0.7 | 1.7 |
| | TaLq93(L) | 24.4±0.7 | | 35.3±2.6 | | 37.7±0.8 | | | 22.6±0.7 | | 34.4±0.6 | | 28.0±1.1 | |
| | | | | | | | | | | | | | | |
| | | | | | | | | | | | | | | |
| II-1 | TaLq1(H) | 42.3±0.8 | 1.5 | 54.5±2.3 | 1.7 | 55.4±1.9 | 1.2 | | 33.1±0.9 | 1.3 | 48.4±1.6 | 1.1 | 41.0±1.2 | 1.1 |
| | TaLq71(L) | 28.7±0.7 | | 32.0±1.0 | | 47.5±1.1 | | | 26.3±0.4 | | 42.5±0.5 | | 36.4±1.6 | |
| | | | | | | | | | | | | | | |
| II-2 | TaLq107(H) | 48.9±1.5 | 1.3 | 52.7±2.6 | 1.4 | 67.0±1.3 | 1.3 | | 32.2±1.6 | 1.1 | 47.6±1.7 | 1.1 | 47.3±0.7 | 1.3 |
| | TaLq58(L) | 38.6±1.4 | | 38.3±1.4 | | 50.3±2.0 | | | 30.5±0.2 | | 44.6±1.0 | | 36.6±0.9 | |
| | | | | | | | | | | | | | | |
| II-3 | TaLq46(H) | 38.7±0.5 | 1.4 | 46.8±0.6 | 1.4 | 52.1±1.5 | 1,2 | | 29.7±0.5 | 1.2 | 43.4±0.8 | 1.2 | 40.8±1.1 | 1.2 |
| | TaLq47(L) | 26.9±1.6 | | 33.8±1.3 | | 41.6±0.8 | | | 23.8±0.2 | | 35.8±0.8 | | 34.2±1.1 | |
| | | | | | | | | | | | | | | |
| | | | | | | | | | | | | | | |
| III-1 | Osfc27(H) | 45.7±1.7 | 1.6 | 59.6±6.3 | 1.9 | 79.7±2.2 | 1.7 | | 55.3±1.6 | 2.1 | 58.4±2.7 | 1.4 | 55.4±1.2 | 1.5 |
| | TaLq71(L) | 28.7±0.7 | | 32.0±1.0 | | 47.5±1.1 | | | 26.3±0.4 | | 42.5±0.5 | | 36.4±1.6 | |
| | | | | | | | | | | | | | | |
| III-2 | Osfc2(H) | 61.1±2.6 | 1.5 | 93.4±4.2 | 1.8 | 100.0±1.6 | 1.7 | | 51.2±1.6 | 1.6 | 61.6±1.4 | 1.2 | 65.4±3.2 | 1.4 |
| | TaLq85(L) | 41.0±0.5 | | 51.3±1.5 | | 58.1±1.8 | | | 31.6±1.3 | | 49.4±2.3 | | 47.1±0.6 | |
| | | | | | | | | | | | | | | |
| III-3 | Osfc32(H) | 51.2±0.9 | 1.3 | 67.4±1.1 | 1.3 | 100.0±1.3 | 1.7 | | 54.8±0.3 | 1.5 | 74.3±2.9 | 1.5 | 68.3±2.7 | 1.7 |
| | TaLq27(L) | 39.2±1.9 | | 51.7±3.0 | | 58.0±1.7 | | | 35.7±1.5 | | 50.3±1.5 | | 40.4±1.1 | |
&, (H) or (L) Indicated the sample in the pair with relatively high (H) or low (L) biomass digestibility;
@, Ratio of two sample values at pair.
