# Supplementary material for: Biomass digestibility is predominantly affected by three factors of wall polymer features distinctive in wheat accessions and rice mutants
Source: Biotechnol Biofuels. 2013 Dec 16;6:183. doi: 10.1186/1754-6834-6-183 (PMC3878626; doi:10.1186/1754-6834-6-183)
Supplement: Additional file 2: Table S2 — Total sugar yield (% cell wall) released from both enzymatic hydrolysis and pretreatment. Exhibited are comparisons of biomass enzymatic digestibility (total hexose and pentose yield) upon sodium hydroxide (NaOH) and sulfuric acid (H2SO4) pretreatments with three concentrations, among a total of nine pairs of wheat and rice samples. [file 1754-6834-6-183-S2.pptx]

## Slide 1
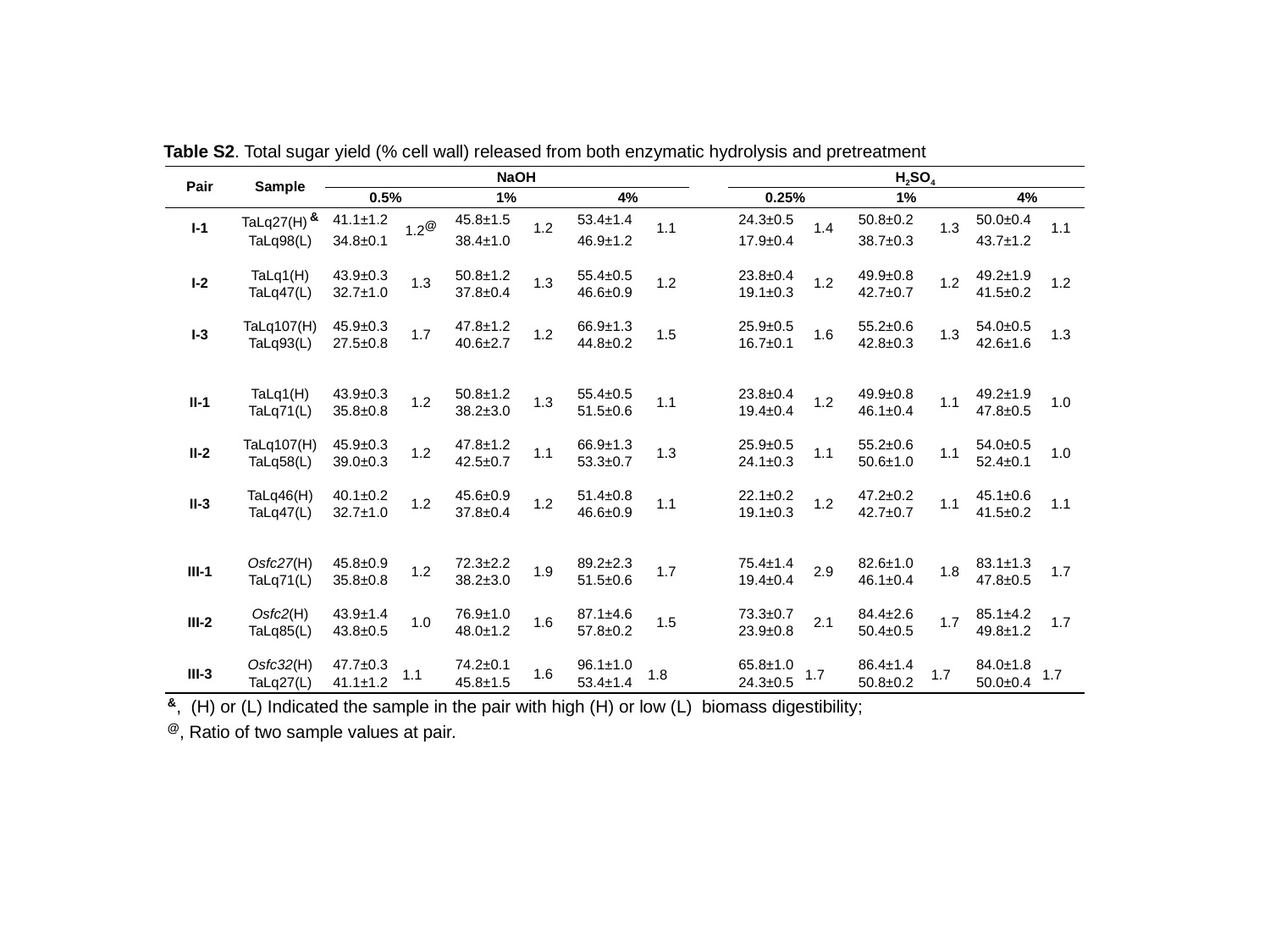

# Table S2. Total sugar yield (% cell wall) released from both enzymatic hydrolysis and pretreatment
| Pair | Sample | NaOH | | | | | | | H2SO4 | | | | | |
| --- | --- | --- | --- | --- | --- | --- | --- | --- | --- | --- | --- | --- | --- | --- |
| | | 0.5% | | 1% | | 4% | | | 0.25% | | 1% | | 4% | |
| I-1 | TaLq27(H) & | 41.1±1.2 | 1.2@ | 45.8±1.5 | 1.2 | 53.4±1.4 | 1.1 | | 24.3±0.5 | 1.4 | 50.8±0.2 | 1.3 | 50.0±0.4 | 1.1 |
| | TaLq98(L) | 34.8±0.1 | | 38.4±1.0 | | 46.9±1.2 | | | 17.9±0.4 | | 38.7±0.3 | | 43.7±1.2 | |
| | | | | | | | | | | | | | | |
| I-2 | TaLq1(H) | 43.9±0.3 | 1.3 | 50.8±1.2 | 1.3 | 55.4±0.5 | 1.2 | | 23.8±0.4 | 1.2 | 49.9±0.8 | 1.2 | 49.2±1.9 | 1.2 |
| | TaLq47(L) | 32.7±1.0 | | 37.8±0.4 | | 46.6±0.9 | | | 19.1±0.3 | | 42.7±0.7 | | 41.5±0.2 | |
| | | | | | | | | | | | | | | |
| I-3 | TaLq107(H) | 45.9±0.3 | 1.7 | 47.8±1.2 | 1.2 | 66.9±1.3 | 1.5 | | 25.9±0.5 | 1.6 | 55.2±0.6 | 1.3 | 54.0±0.5 | 1.3 |
| | TaLq93(L) | 27.5±0.8 | | 40.6±2.7 | | 44.8±0.2 | | | 16.7±0.1 | | 42.8±0.3 | | 42.6±1.6 | |
| | | | | | | | | | | | | | | |
| | | | | | | | | | | | | | | |
| II-1 | TaLq1(H) | 43.9±0.3 | 1.2 | 50.8±1.2 | 1.3 | 55.4±0.5 | 1.1 | | 23.8±0.4 | 1.2 | 49.9±0.8 | 1.1 | 49.2±1.9 | 1.0 |
| | TaLq71(L) | 35.8±0.8 | | 38.2±3.0 | | 51.5±0.6 | | | 19.4±0.4 | | 46.1±0.4 | | 47.8±0.5 | |
| | | | | | | | | | | | | | | |
| II-2 | TaLq107(H) | 45.9±0.3 | 1.2 | 47.8±1.2 | 1.1 | 66.9±1.3 | 1.3 | | 25.9±0.5 | 1.1 | 55.2±0.6 | 1.1 | 54.0±0.5 | 1.0 |
| | TaLq58(L) | 39.0±0.3 | | 42.5±0.7 | | 53.3±0.7 | | | 24.1±0.3 | | 50.6±1.0 | | 52.4±0.1 | |
| | | | | | | | | | | | | | | |
| II-3 | TaLq46(H) | 40.1±0.2 | 1.2 | 45.6±0.9 | 1.2 | 51.4±0.8 | 1.1 | | 22.1±0.2 | 1.2 | 47.2±0.2 | 1.1 | 45.1±0.6 | 1.1 |
| | TaLq47(L) | 32.7±1.0 | | 37.8±0.4 | | 46.6±0.9 | | | 19.1±0.3 | | 42.7±0.7 | | 41.5±0.2 | |
| | | | | | | | | | | | | | | |
| | | | | | | | | | | | | | | |
| III-1 | Osfc27(H) | 45.8±0.9 | 1.2 | 72.3±2.2 | 1.9 | 89.2±2.3 | 1.7 | | 75.4±1.4 | 2.9 | 82.6±1.0 | 1.8 | 83.1±1.3 | 1.7 |
| | TaLq71(L) | 35.8±0.8 | | 38.2±3.0 | | 51.5±0.6 | | | 19.4±0.4 | | 46.1±0.4 | | 47.8±0.5 | |
| | | | | | | | | | | | | | | |
| III-2 | Osfc2(H) | 43.9±1.4 | 1.0 | 76.9±1.0 | 1.6 | 87.1±4.6 | 1.5 | | 73.3±0.7 | 2.1 | 84.4±2.6 | 1.7 | 85.1±4.2 | 1.7 |
| | TaLq85(L) | 43.8±0.5 | | 48.0±1.2 | | 57.8±0.2 | | | 23.9±0.8 | | 50.4±0.5 | | 49.8±1.2 | |
| | | | | | | | | | | | | | | |
| III-3 | Osfc32(H) | 47.7±0.3 | 1.1 | 74.2±0.1 | 1.6 | 96.1±1.0 | 1.8 | | 65.8±1.0 | 1.7 | 86.4±1.4 | 1.7 | 84.0±1.8 | 1.7 |
| | TaLq27(L) | 41.1±1.2 | | 45.8±1.5 | | 53.4±1.4 | | | 24.3±0.5 | | 50.8±0.2 | | 50.0±0.4 | |
&, (H) or (L) Indicated the sample in the pair with high (H) or low (L) biomass digestibility;
@, Ratio of two sample values at pair.
