# Supplementary material for: Biomass digestibility is predominantly affected by three factors of wall polymer features distinctive in wheat accessions and rice mutants
Source: Biotechnol Biofuels. 2013 Dec 16;6:183. doi: 10.1186/1754-6834-6-183 (PMC3878626; doi:10.1186/1754-6834-6-183)
Supplement: Additional file 3: Table S3 — The crystalline index (CrI) of raw materials in wheat and rice samples. Displayed are comparisons of CrI values among a total of nine pairs of wheat and rice samples. [file 1754-6834-6-183-S3.pptx]

## Slide 1
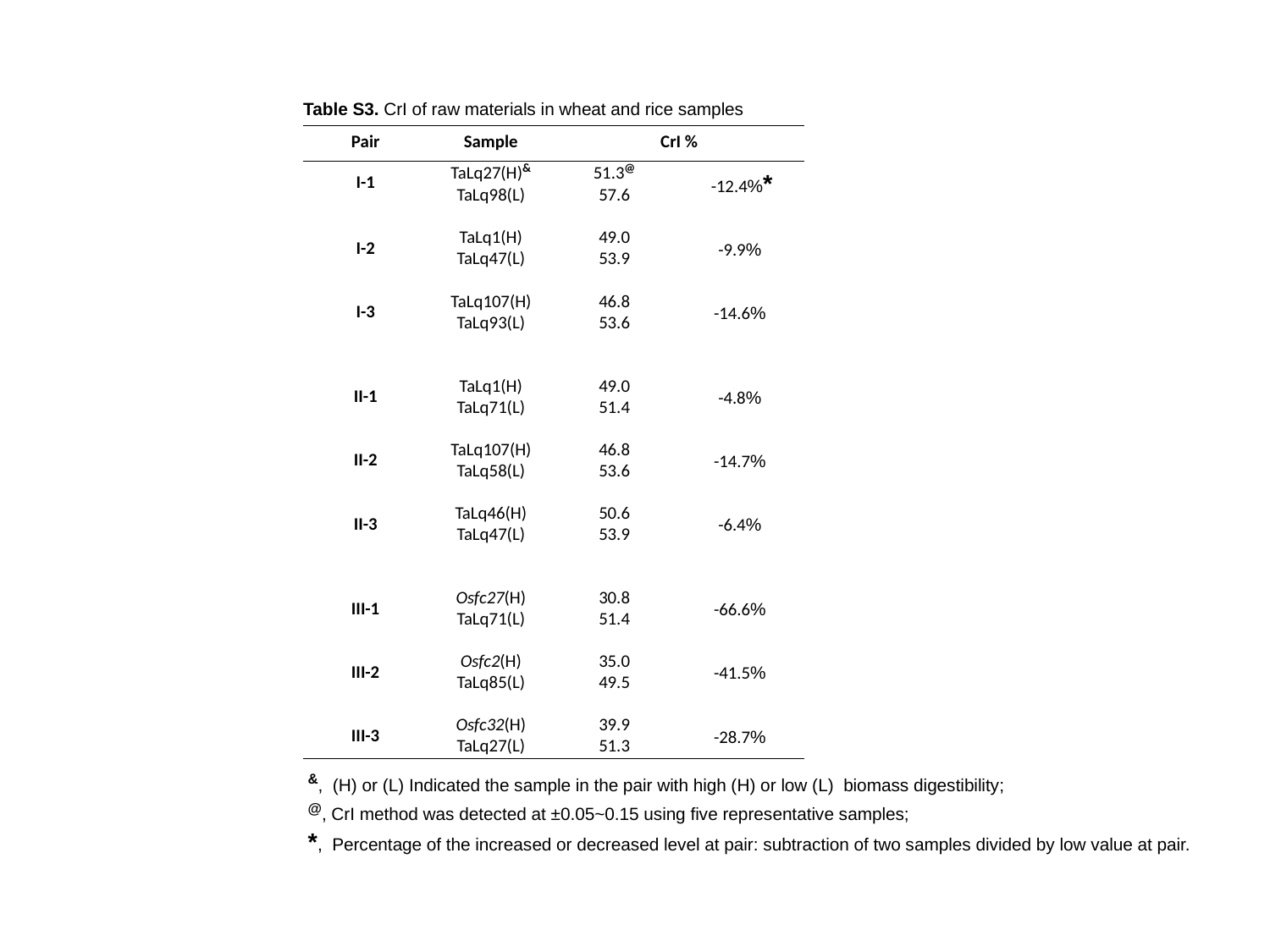

# Table S3. CrI of raw materials in wheat and rice samples
| Pair | Sample | CrI % | |
| --- | --- | --- | --- |
| I-1 | TaLq27(H)& | 51.3@ | -12.4%\* |
| | TaLq98(L) | 57.6 | |
| | | | |
| I-2 | TaLq1(H) | 49.0 | -9.9% |
| | TaLq47(L) | 53.9 | |
| | | | |
| I-3 | TaLq107(H) | 46.8 | -14.6% |
| | TaLq93(L) | 53.6 | |
| | | | |
| | | | |
| II-1 | TaLq1(H) | 49.0 | -4.8% |
| | TaLq71(L) | 51.4 | |
| | | | |
| II-2 | TaLq107(H) | 46.8 | -14.7% |
| | TaLq58(L) | 53.6 | |
| | | | |
| II-3 | TaLq46(H) | 50.6 | -6.4% |
| | TaLq47(L) | 53.9 | |
| | | | |
| | | | |
| III-1 | Osfc27(H) | 30.8 | -66.6% |
| | TaLq71(L) | 51.4 | |
| | | | |
| III-2 | Osfc2(H) | 35.0 | -41.5% |
| | TaLq85(L) | 49.5 | |
| | | | |
| III-3 | Osfc32(H) | 39.9 | -28.7% |
| | TaLq27(L) | 51.3 | |
&, (H) or (L) Indicated the sample in the pair with high (H) or low (L) biomass digestibility;
@, CrI method was detected at ±0.05~0.15 using five representative samples;
*, Percentage of the increased or decreased level at pair: subtraction of two samples divided by low value at pair.
