# Supplementary material for: Biomass digestibility is predominantly affected by three factors of wall polymer features distinctive in wheat accessions and rice mutants
Source: Biotechnol Biofuels. 2013 Dec 16;6:183. doi: 10.1186/1754-6834-6-183 (PMC3878626; doi:10.1186/1754-6834-6-183)
Supplement: Additional file 4: Table S4 — Monosaccharide composition of hemicelluloses. Displayed are comparisons of monosaccharide compositions in the potassium hydroxide (KOH)-extractable and non-KOH-extractable hemicelluloses among a total of nine pairs of wheat and rice samples. [file 1754-6834-6-183-S4.pptx]

## Slide 1
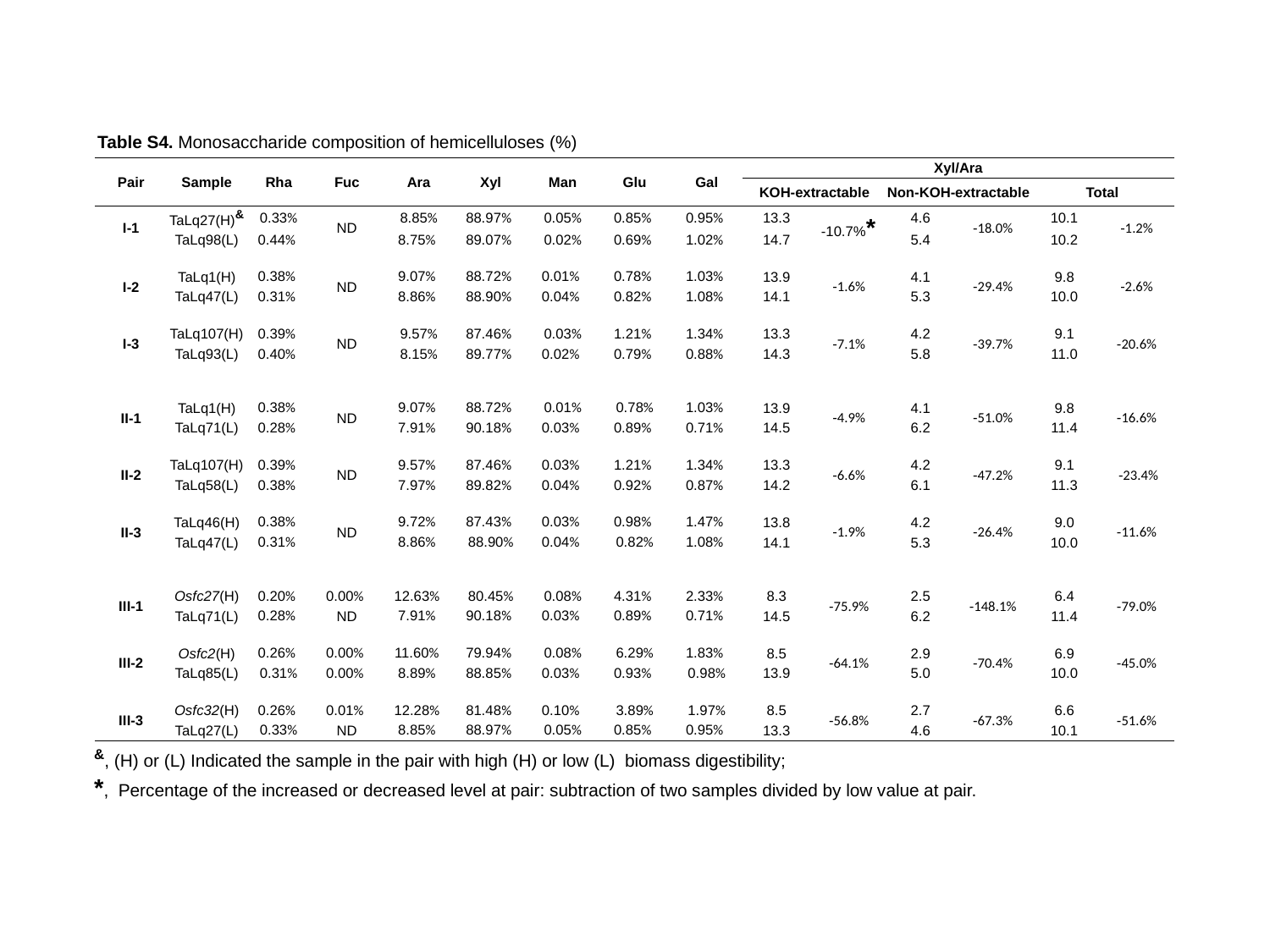

Table S4. Monosaccharide composition of hemicelluloses (%)
| Pair | Sample | Rha | Fuc | Ara | Xyl | Man | Glu | Gal | Xyl/Ara | | | | | |
| --- | --- | --- | --- | --- | --- | --- | --- | --- | --- | --- | --- | --- | --- | --- |
| | | | | | | | | | KOH-extractable | | Non-KOH-extractable | | Total | |
| I-1 | TaLq27(H)& | 0.33% | ND | 8.85% | 88.97% | 0.05% | 0.85% | 0.95% | 13.3 | -10.7%\* | 4.6 | -18.0% | 10.1 | -1.2% |
| | TaLq98(L) | 0.44% | | 8.75% | 89.07% | 0.02% | 0.69% | 1.02% | 14.7 | | 5.4 | | 10.2 | |
| | | | | | | | | | | | | | | |
| I-2 | TaLq1(H) | 0.38% | ND | 9.07% | 88.72% | 0.01% | 0.78% | 1.03% | 13.9 | -1.6% | 4.1 | -29.4% | 9.8 | -2.6% |
| | TaLq47(L) | 0.31% | | 8.86% | 88.90% | 0.04% | 0.82% | 1.08% | 14.1 | | 5.3 | | 10.0 | |
| | | | | | | | | | | | | | | |
| I-3 | TaLq107(H) | 0.39% | ND | 9.57% | 87.46% | 0.03% | 1.21% | 1.34% | 13.3 | -7.1% | 4.2 | -39.7% | 9.1 | -20.6% |
| | TaLq93(L) | 0.40% | | 8.15% | 89.77% | 0.02% | 0.79% | 0.88% | 14.3 | | 5.8 | | 11.0 | |
| | | | | | | | | | | | | | | |
| | | | | | | | | | | | | | | |
| II-1 | TaLq1(H) | 0.38% | ND | 9.07% | 88.72% | 0.01% | 0.78% | 1.03% | 13.9 | -4.9% | 4.1 | -51.0% | 9.8 | -16.6% |
| | TaLq71(L) | 0.28% | | 7.91% | 90.18% | 0.03% | 0.89% | 0.71% | 14.5 | | 6.2 | | 11.4 | |
| | | | | | | | | | | | | | | |
| II-2 | TaLq107(H) | 0.39% | ND | 9.57% | 87.46% | 0.03% | 1.21% | 1.34% | 13.3 | -6.6% | 4.2 | -47.2% | 9.1 | -23.4% |
| | TaLq58(L) | 0.38% | | 7.97% | 89.82% | 0.04% | 0.92% | 0.87% | 14.2 | | 6.1 | | 11.3 | |
| | | | | | | | | | | | | | | |
| II-3 | TaLq46(H) | 0.38% | ND | 9.72% | 87.43% | 0.03% | 0.98% | 1.47% | 13.8 | -1.9% | 4.2 | -26.4% | 9.0 | -11.6% |
| | TaLq47(L) | 0.31% | | 8.86% | 88.90% | 0.04% | 0.82% | 1.08% | 14.1 | | 5.3 | | 10.0 | |
| | | | | | | | | | | | | | | |
| | | | | | | | | | | | | | | |
| III-1 | Osfc27(H) | 0.20% | 0.00% | 12.63% | 80.45% | 0.08% | 4.31% | 2.33% | 8.3 | -75.9% | 2.5 | -148.1% | 6.4 | -79.0% |
| | TaLq71(L) | 0.28% | ND | 7.91% | 90.18% | 0.03% | 0.89% | 0.71% | 14.5 | | 6.2 | | 11.4 | |
| | | | | | | | | | | | | | | |
| III-2 | Osfc2(H) | 0.26% | 0.00% | 11.60% | 79.94% | 0.08% | 6.29% | 1.83% | 8.5 | -64.1% | 2.9 | -70.4% | 6.9 | -45.0% |
| | TaLq85(L) | 0.31% | 0.00% | 8.89% | 88.85% | 0.03% | 0.93% | 0.98% | 13.9 | | 5.0 | | 10.0 | |
| | | | | | | | | | | | | | | |
| III-3 | Osfc32(H) | 0.26% | 0.01% | 12.28% | 81.48% | 0.10% | 3.89% | 1.97% | 8.5 | -56.8% | 2.7 | -67.3% | 6.6 | -51.6% |
| | TaLq27(L) | 0.33% | ND | 8.85% | 88.97% | 0.05% | 0.85% | 0.95% | 13.3 | | 4.6 | | 10.1 | |
&, (H) or (L) Indicated the sample in the pair with high (H) or low (L) biomass digestibility;
*, Percentage of the increased or decreased level at pair: subtraction of two samples divided by low value at pair.
