# Supplementary material for: Biomass digestibility is predominantly affected by three factors of wall polymer features distinctive in wheat accessions and rice mutants
Source: Biotechnol Biofuels. 2013 Dec 16;6:183. doi: 10.1186/1754-6834-6-183 (PMC3878626; doi:10.1186/1754-6834-6-183)
Supplement: Additional file 5: Table S5 — Ratios of three monolignins. Displayed are comparisons of three monomer ratios in the potassium hydroxide (KOH)-extractable and non-KOH-extractable lignin among a total of nine pairs of wheat and rice samples. [file 1754-6834-6-183-S5.pptx]

## Slide 1
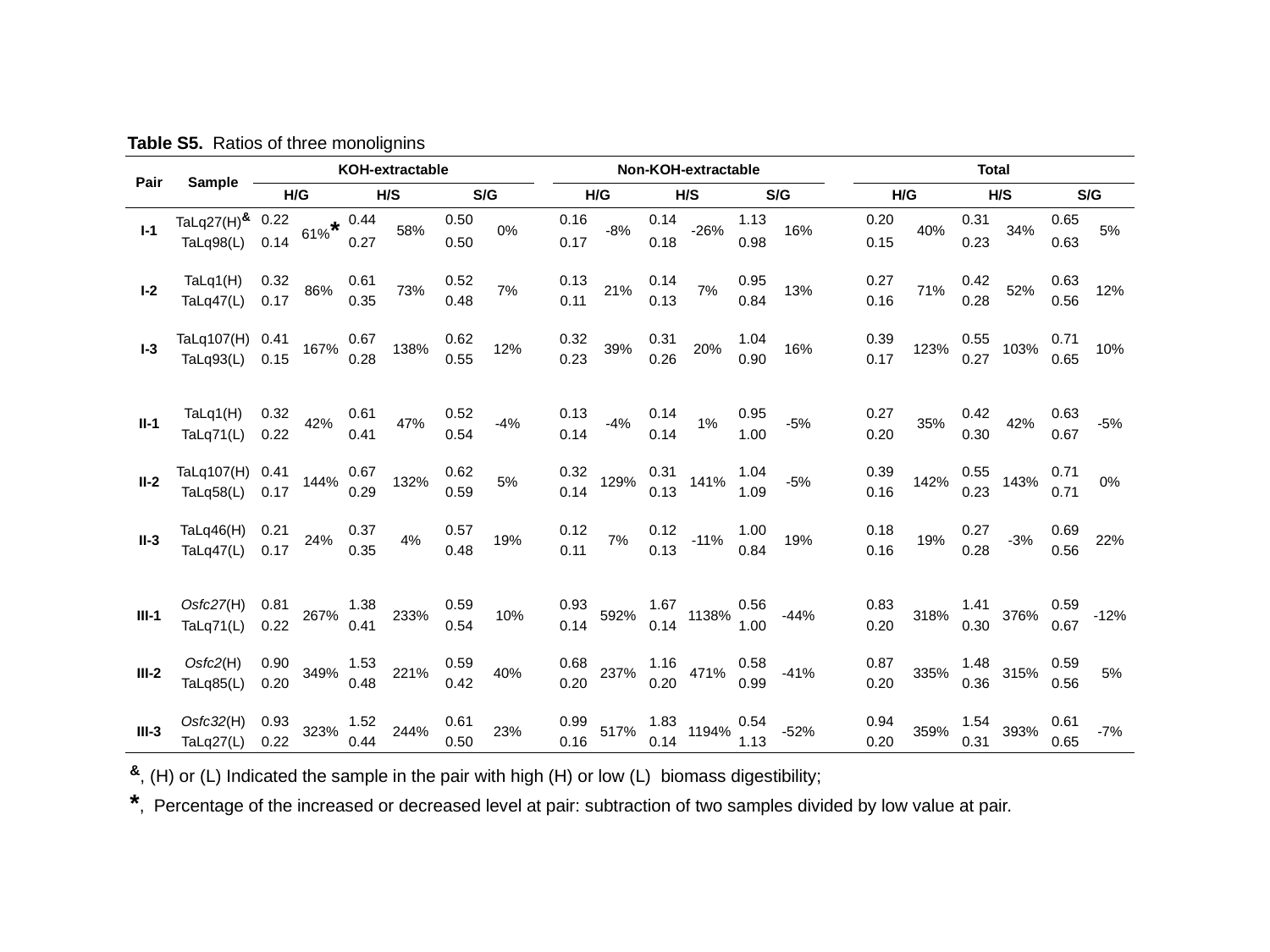

# Table S5. Ratios of three monolignins
| Pair | Sample | KOH-extractable | | | | | | | Non-KOH-extractable | | | | | | | Total | | | | | |
| --- | --- | --- | --- | --- | --- | --- | --- | --- | --- | --- | --- | --- | --- | --- | --- | --- | --- | --- | --- | --- | --- |
| | | H/G | | H/S | | S/G | | | H/G | | H/S | | S/G | | | H/G | | H/S | | S/G | |
| I-1 | TaLq27(H)& | 0.22 | 61%\* | 0.44 | 58% | 0.50 | 0% | | 0.16 | -8% | 0.14 | -26% | 1.13 | 16% | | 0.20 | 40% | 0.31 | 34% | 0.65 | 5% |
| | TaLq98(L) | 0.14 | | 0.27 | | 0.50 | | | 0.17 | | 0.18 | | 0.98 | | | 0.15 | | 0.23 | | 0.63 | |
| | | | | | | | | | | | | | | | | | | | | | |
| I-2 | TaLq1(H) | 0.32 | 86% | 0.61 | 73% | 0.52 | 7% | | 0.13 | 21% | 0.14 | 7% | 0.95 | 13% | | 0.27 | 71% | 0.42 | 52% | 0.63 | 12% |
| | TaLq47(L) | 0.17 | | 0.35 | | 0.48 | | | 0.11 | | 0.13 | | 0.84 | | | 0.16 | | 0.28 | | 0.56 | |
| | | | | | | | | | | | | | | | | | | | | | |
| I-3 | TaLq107(H) | 0.41 | 167% | 0.67 | 138% | 0.62 | 12% | | 0.32 | 39% | 0.31 | 20% | 1.04 | 16% | | 0.39 | 123% | 0.55 | 103% | 0.71 | 10% |
| | TaLq93(L) | 0.15 | | 0.28 | | 0.55 | | | 0.23 | | 0.26 | | 0.90 | | | 0.17 | | 0.27 | | 0.65 | |
| | | | | | | | | | | | | | | | | | | | | | |
| | | | | | | | | | | | | | | | | | | | | | |
| II-1 | TaLq1(H) | 0.32 | 42% | 0.61 | 47% | 0.52 | -4% | | 0.13 | -4% | 0.14 | 1% | 0.95 | -5% | | 0.27 | 35% | 0.42 | 42% | 0.63 | -5% |
| | TaLq71(L) | 0.22 | | 0.41 | | 0.54 | | | 0.14 | | 0.14 | | 1.00 | | | 0.20 | | 0.30 | | 0.67 | |
| | | | | | | | | | | | | | | | | | | | | | |
| II-2 | TaLq107(H) | 0.41 | 144% | 0.67 | 132% | 0.62 | 5% | | 0.32 | 129% | 0.31 | 141% | 1.04 | -5% | | 0.39 | 142% | 0.55 | 143% | 0.71 | 0% |
| | TaLq58(L) | 0.17 | | 0.29 | | 0.59 | | | 0.14 | | 0.13 | | 1.09 | | | 0.16 | | 0.23 | | 0.71 | |
| | | | | | | | | | | | | | | | | | | | | | |
| II-3 | TaLq46(H) | 0.21 | 24% | 0.37 | 4% | 0.57 | 19% | | 0.12 | 7% | 0.12 | -11% | 1.00 | 19% | | 0.18 | 19% | 0.27 | -3% | 0.69 | 22% |
| | TaLq47(L) | 0.17 | | 0.35 | | 0.48 | | | 0.11 | | 0.13 | | 0.84 | | | 0.16 | | 0.28 | | 0.56 | |
| | | | | | | | | | | | | | | | | | | | | | |
| | | | | | | | | | | | | | | | | | | | | | |
| III-1 | Osfc27(H) | 0.81 | 267% | 1.38 | 233% | 0.59 | 10% | | 0.93 | 592% | 1.67 | 1138% | 0.56 | -44% | | 0.83 | 318% | 1.41 | 376% | 0.59 | -12% |
| | TaLq71(L) | 0.22 | | 0.41 | | 0.54 | | | 0.14 | | 0.14 | | 1.00 | | | 0.20 | | 0.30 | | 0.67 | |
| | | | | | | | | | | | | | | | | | | | | | |
| III-2 | Osfc2(H) | 0.90 | 349% | 1.53 | 221% | 0.59 | 40% | | 0.68 | 237% | 1.16 | 471% | 0.58 | -41% | | 0.87 | 335% | 1.48 | 315% | 0.59 | 5% |
| | TaLq85(L) | 0.20 | | 0.48 | | 0.42 | | | 0.20 | | 0.20 | | 0.99 | | | 0.20 | | 0.36 | | 0.56 | |
| | | | | | | | | | | | | | | | | | | | | | |
| III-3 | Osfc32(H) | 0.93 | 323% | 1.52 | 244% | 0.61 | 23% | | 0.99 | 517% | 1.83 | 1194% | 0.54 | -52% | | 0.94 | 359% | 1.54 | 393% | 0.61 | -7% |
| | TaLq27(L) | 0.22 | | 0.44 | | 0.50 | | | 0.16 | | 0.14 | | 1.13 | | | 0.20 | | 0.31 | | 0.65 | |
&, (H) or (L) Indicated the sample in the pair with high (H) or low (L) biomass digestibility;
*, Percentage of the increased or decreased level at pair: subtraction of two samples divided by low value at pair.
