# Supplementary material for: Biomass digestibility is predominantly affected by three factors of wall polymer features distinctive in wheat accessions and rice mutants
Source: Biotechnol Biofuels. 2013 Dec 16;6:183. doi: 10.1186/1754-6834-6-183 (PMC3878626; doi:10.1186/1754-6834-6-183)
Supplement: Additional file 6: Table S6 — Variation of two types of hemicellulose (μmol/g dry matter). Exhibited are proportions between the potassium hydroxide (KOH)-extractable and non-KOH-extractable hemicelluloses in the representative wheat (n = 10) and rice (n = 3) samples. [file 1754-6834-6-183-S6.pptx]

## Slide 1
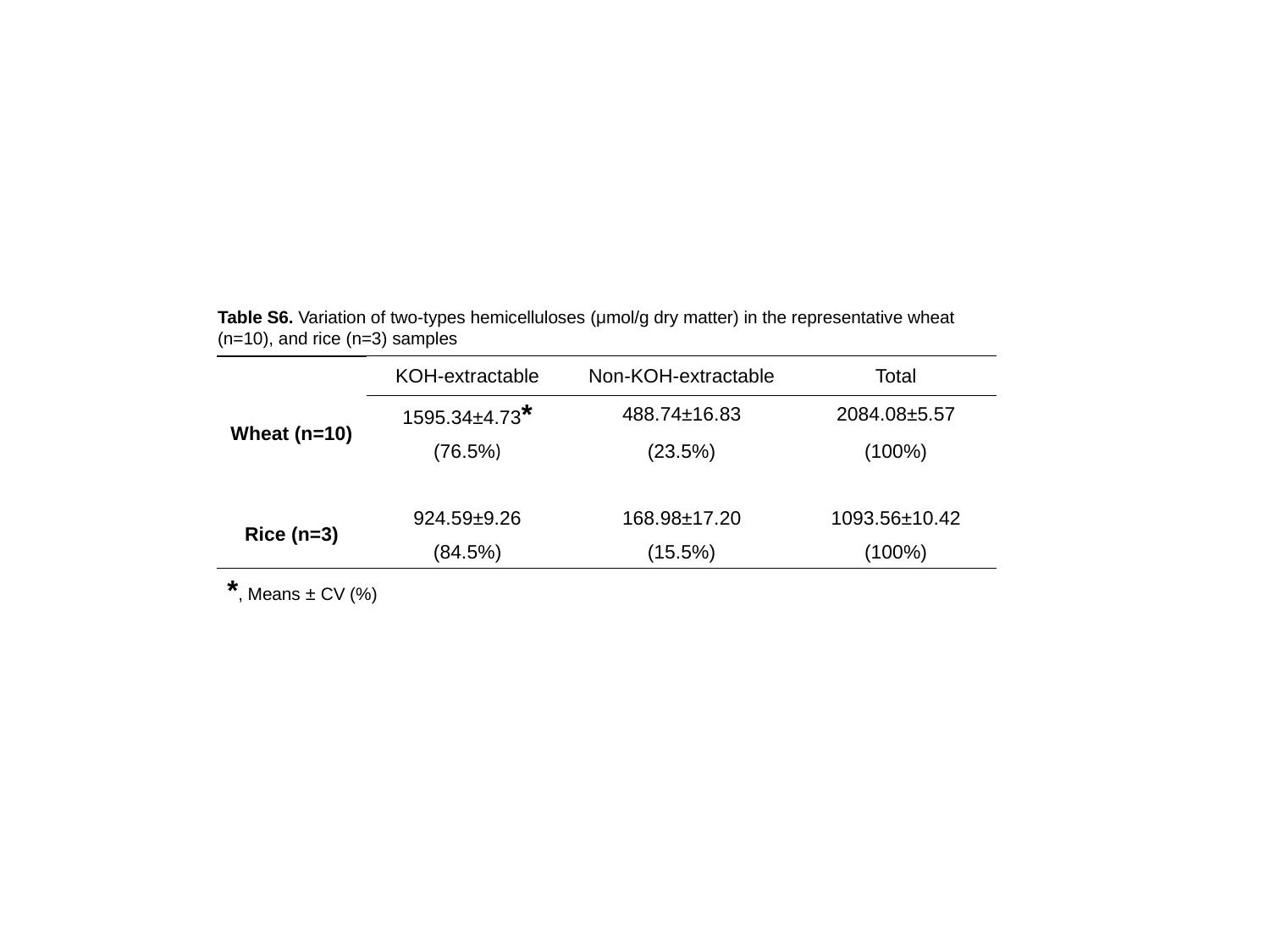

Table S6. Variation of two-types hemicelluloses (μmol/g dry matter) in the representative wheat (n=10), and rice (n=3) samples
| | KOH-extractable | Non-KOH-extractable | Total |
| --- | --- | --- | --- |
| Wheat (n=10) | 1595.34±4.73\* | 488.74±16.83 | 2084.08±5.57 |
| | (76.5%) | (23.5%) | (100%) |
| | | | |
| Rice (n=3) | 924.59±9.26 | 168.98±17.20 | 1093.56±10.42 |
| | (84.5%) | (15.5%) | (100%) |
*, Means ± CV (%)
