# Supplementary material for: Biomass digestibility is predominantly affected by three factors of wall polymer features distinctive in wheat accessions and rice mutants
Source: Biotechnol Biofuels. 2013 Dec 16;6:183. doi: 10.1186/1754-6834-6-183 (PMC3878626; doi:10.1186/1754-6834-6-183)
Supplement: Additional file 7: Table S7 — Variation of two types of lignin (μmol/g dry matter). Exhibited proportions between the potassium hydroxide (KOH)-extractable and non-KOH-extractable lignin in the representative wheat (n = 10) and rice (n = 3) samples. [file 1754-6834-6-183-S7.pptx]

## Slide 1
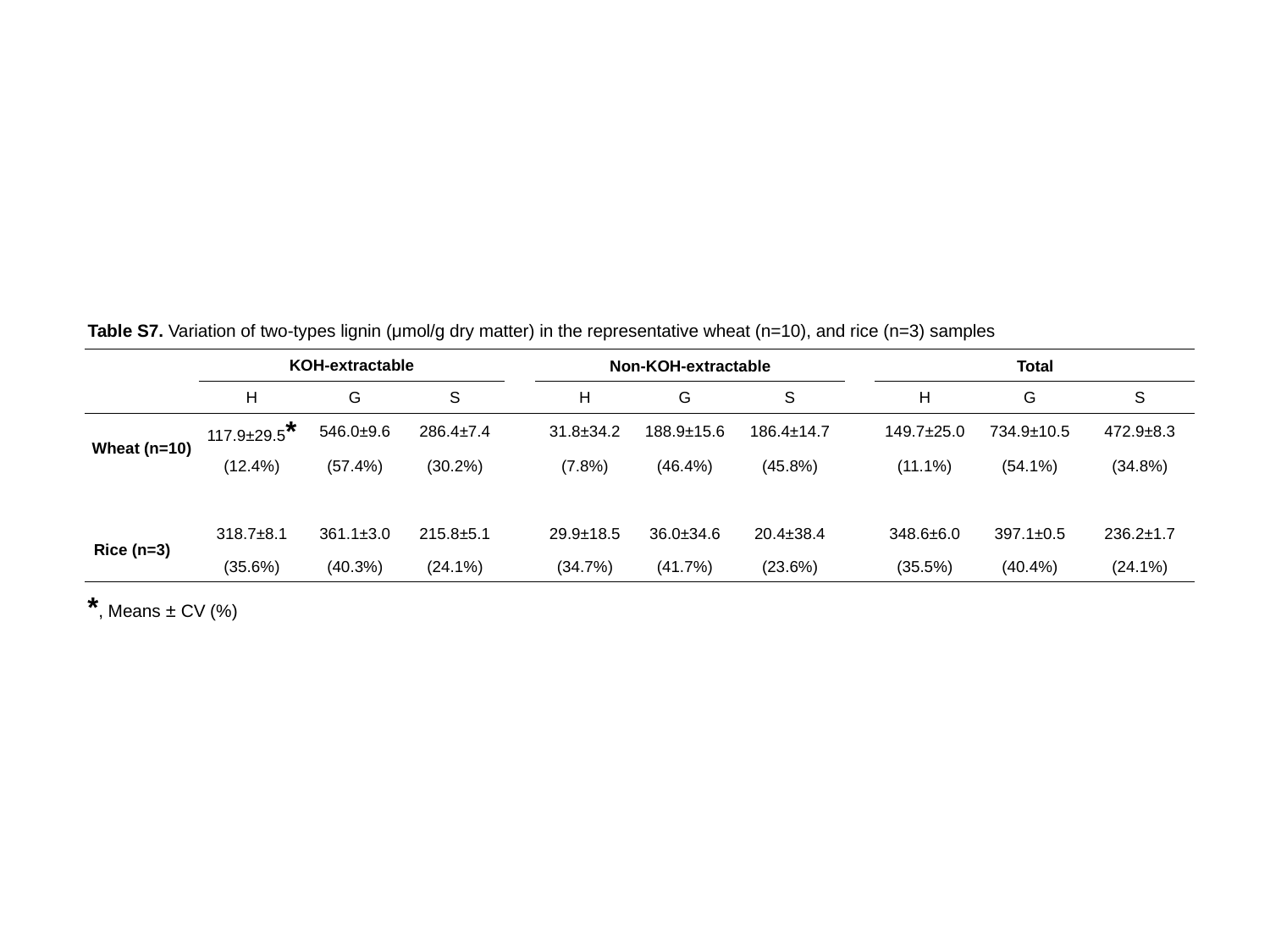

Table S7. Variation of two-types lignin (μmol/g dry matter) in the representative wheat (n=10), and rice (n=3) samples
| | KOH-extractable | | | | Non-KOH-extractable | | | | Total | | |
| --- | --- | --- | --- | --- | --- | --- | --- | --- | --- | --- | --- |
| | H | G | S | | H | G | S | | H | G | S |
| Wheat (n=10) | 117.9±29.5\* | 546.0±9.6 | 286.4±7.4 | | 31.8±34.2 | 188.9±15.6 | 186.4±14.7 | | 149.7±25.0 | 734.9±10.5 | 472.9±8.3 |
| | (12.4%) | (57.4%) | (30.2%) | | (7.8%) | (46.4%) | (45.8%) | | (11.1%) | (54.1%) | (34.8%) |
| | | | | | | | | | | | |
| Rice (n=3) | 318.7±8.1 | 361.1±3.0 | 215.8±5.1 | | 29.9±18.5 | 36.0±34.6 | 20.4±38.4 | | 348.6±6.0 | 397.1±0.5 | 236.2±1.7 |
| | (35.6%) | (40.3%) | (24.1%) | | (34.7%) | (41.7%) | (23.6%) | | (35.5%) | (40.4%) | (24.1%) |
*, Means ± CV (%)
